# Associations between trajectories of cardiovascular risk factor change and cognitive impairment in Chinese elderly: A nationwide cohort study

**DOI:** 10.3389/fnagi.2023.1084136

**Published:** 2023-02-10

**Authors:** Xinyu Duan, Yusong Dang, Chenxi Kang, Peixi Rong, Mingxin Yan, Shutong Zhang, Jing Cui, Yaling Zhao, Fangyao Chen, Jing Zhou, Duolao Wang, Leilei Pei

**Affiliations:** ^1^Department of Epidemiology and Health Statistics, School of Public Health, Xi’an Jiaotong University Health Science Center, Xi’an, Shaanxi, China; ^2^Department of Pediatrics, The Second Affiliated Hospital of Xi’an Jiaotong University, Xi’an, Shaanxi, China; ^3^Biostatistics Unit, Department of Clinical Sciences, Liverpool School of Tropical Medicine, Pembroke Place, Liverpool, United Kingdom; ^4^Department of Neurology, Guangdong Key Laboratory of Age-Related Cardiac and Cerebral Diseases, Affiliated Hospital of Guangdong Medical University, Zhanjiang, China

**Keywords:** trajectory, cardiovascular risk factors, cognitive impairment, Chinese elderly, blood pressure, BMI

## Abstract

**Objectives:**

This study aimed to investigate the relationship between long-term trajectories of changes in cardiovascular risk factors (CVRFs) and the risk of cognitive impairment among Chinese adults over 60 years old.

**Methods:**

Data were obtained from the Chinese Longitudinal Healthy Longevity Survey 2005–2018. Cognitive function was evaluated longitudinally through the Chinese version of the Mini-Mental State Examination (C-MMSE), and cognitive impairment (C-MMSE ≤23) was used as the main outcome variable. The cardiovascular risk factors, including systolic blood pressure (SBP), diastolic blood pressure (DBP), mean arterial pressure (MAP), pulse pressure (PP), and body mass index (BMI), were continuously measured in the follow-up duration. The patterns of trajectories of changes in CVRFs were derived from the latent growth mixture model (LGMM). The Cox regression model was used to evaluate the cognitive impairment hazard ratio (HR) across different CVRF trajectories.

**Results:**

A total of 5,164 participants aged ≥60 years with normal cognitive function at baseline were included in the study. After a median follow-up of 8 years, 2,071 participants (40.1%) developed cognitive impairment (C-MMSE ≤ 23). The four-class trajectories of SBP and BMI were obtained by means of LGMM, and the trajectories of DBP, MAP, and PP were grouped into a three-class subgroup. In the final adjusted Cox model, the lowered SBP [adjusted HR (aHR): 1.59; 95% CI: 1.17–2.16], lowered PP (aHR: 2.64; 95% CI: 1.66–4.19), and progressively obese (aHR: 1.28; 95% CI: 1.02–1.62) and stable slim (aHR: 1.13; 95% CI: 1.02–1.25) were associated with the higher risk of cognitive impairment. Low stable DBP (aHR: 0.80; 95% CI: 0.66–0.96) and elevated PP (aHR: 0.76; 95% CI: 0.63–0.92) decreased the risk for cognitive impairment among participants.

**Conclusion:**

Lowered SBP, lowered PP, progressive obesity, and stable slim increased the risk for cognitive impairment in the Chinese elderly. Low stable DBP and elevated PP were protective against cognitive impairment, but more DBP lowering and ≥25 mmHg growth in PP contributed to a higher risk of cognitive impairment. The findings have important implications for preventing cognitive impairment in elder adults based on the long-term trajectories of changes in CVRFs.

## 1. Introduction

With the increase in human lifespan, dementia imposes a heavy burden on families, society, and health-care systems (Yu et al., 2012). It is reported that the global dementia population reached 47 million in 2015 and will rise to 131 million by 2050 (Arvanitakis et al., 2019). Cognitive impairment, characterized by declines in memory, attention, and cognitive function, is a transitional state between normal aging and dementia (Gao et al., 2017). A previous study has shown that 10%–15% of people with mild cognitive impairment could develop dementia each year (Eshkoor et al., 2015). Therefore, delaying the onset of cognitive impairment may be a prospective clinical strategy to prevent dementia (Gao et al., 2017). Previous studies have recognized that age, gender, educational level, cardiovascular risk factors (CVRFs), family history, and APOE4 allele are the risk factors for cognitive impairment (Beeri et al., 2009; Zhang et al., 2019; Livingston et al., 2020). Among them, CVRFs have attracted more and more researchers’ attention because of their potentially modifiable features.

Researchers have found that diabetes is strongly associated with the development of cognitive impairment in the elderly population, whereas the findings for other risk factors are more mixed (Kerola et al., 2011). For example, some studies report elevated blood pressure prior to the onset of mild cognitive impairment or dementia, while others show that low blood pressure is associated with an increased risk for cognitive impairment (Launer et al., 2000; Kivipelto et al., 2001; Qiu et al., 2003; Reitz et al., 2007; Lv et al., 2017). The association between obesity and cognitive impairment is also controversial (Wanleenuwat et al., 2019). Some studies showed that being underweight and substantial weight loss are important risk factors for cognitive impairment in the elderly (Ren et al., 2021; Wu et al., 2021), but others suggested that obesity might impair cognitive function, especially when coexisting with other CVRFs (Gustafson and Luchsinger, 2013; Wang et al., 2019). Therefore, the relationships between CVRFs and cognitive impairment need to be further explored.

Previous studies have mainly explored the relationships between CVRFs at a certain moment and the subsequent onset of cognitive impairment (Launer et al., 2000; Kivipelto et al., 2001; Qiu et al., 2003; Reitz et al., 2007; Lv et al., 2017; Wang et al., 2019; Ren et al., 2021; Wu et al., 2021). However, it is acknowledged that CVRFs are not static but change with age during the life course, so the associations of CVRFs with cognitive impairment could alter over time (Walker et al., 2019). Thus, it is more meaningful to analyze the trajectories of changes in CVRFs over time to show the direction and magnitude of changes in CVRFs during the life course (Cheng et al., 2021). The latent growth mixed model (LGMM) can identify multiple unobserved subpopulations that respond similarly to repeated measures of CVRFs (Qiu et al., 2020) and describe the developmental trajectories of subpopulations over time. Given the strong association between cardiovascular burden and cognitive impairment (Beeri et al., 2009; Kerola et al., 2011; Cleveland, 2020), we hypothesized that the patterns of trajectories of changes in CVRFs are predictive of cognitive impairment.

As a populous country, approximately 260 million people aged 60 and above lived in China in 2020, with the elderly population expected to increase to one-third by 2050 (Zhang et al., 2022). Inevitably, with severe aging, the growth rate of people with cognitive impairment in China is the highest in the world due to growing exposure to CVRFs (Wu et al., 2013; Prince et al., 2016). However, most studies investigating the relationships between the trajectories of CVRFs and cognitive impairment were conducted in developed countries (Hakala et al., 2021; Yaffe et al., 2021), with only a few in China. Therefore, based on a national cohort of adults over 60 in China, the study aimed to determine trajectories of changes in CVRFs using the LGMM and explore the relationships between trajectories of changes in CVRFs and cognitive impairment.

## 2. Methods

### 2.1. Participants

The study data were derived from the Chinese Longitudinal Healthy Longevity Survey (CLHLS), an ongoing longitudinal cohort jointly established by Peking University and Duke University. To investigate the determinants of the health of the Chinese elderly, the CLHLS began in 1998 and was followed in 2000, 2002, 2005, 2008–2009, 2011–2012, 2014, and 2017–2018. The first eight waves of survey covered about half of the counties/cities in 23 China provinces, and the ongoing ninth wave of survey further expanded to 27 provinces. To compensate for missing samples due to death and loss to follow-up in the ongoing survey, each wave of surveys continually recruits new participants based on similar gender, age, and baseline characteristics (Zeng, 2012). CLHLS procedures were approved by the Ethics Committee of Peking University and Duke University (IRB00001052-13074). All participants provided written informed consent. A more detailed description of the design and procedures of CLHLS has been described elsewhere (Yi, 2008).

Considering the lack of height data in the first three waves of survey (1998, 2000, and 2002) and fewer than three follow-up visits in the last two waves (2014 and 2018); in the study, we included the participants who were enrolled in the three surveys conducted in 2005, 2008–2009, and 2011–2012 and followed up to 2018. As summarized in Supplementary Figure S1, a total of 26,477 participants were extracted from the three cohorts. However, 19,375 participants were excluded because of fewer than three visits during the follow-up, and 1,925 participants were excluded because they were diagnosed with dementia or cognitive impairment at the baseline. Among the remaining 5,177 participants, 8 participants aged <60 years at the baseline and 5 participants without assessment of cognitive impairment at the last visit were also excluded. Finally, a total of 5,164 participants were included in the study.

### 2.2. Assessment of cognitive function

Cognitive function was evaluated using the Chinese version of the Mini-Mental State Examination (C-MMSE), which was a modified and validated cognitive scale for the China elderly (Zeng, 2012; Ming-yue et al., 2015; Gao et al., 2017). The C-MMSE scale consists of 6 dimensions with 24 items, including 5 items for orientation, 1 for naming, 3 for registration, 6 for attention and calculation, 3 for recall, and 6 for language. The total score of the C-MMSE ranges from 0 to 30, with lower scores indicating poorer cognitive function.

According to the definition of cognitive impairment (Ming-yue et al., 2015; An and Liu, 2016), we classified cognitive functions into four mutually exclusive groups: (1) 24 ≤ C-MMSE ≤30: no cognitive impairment; (2) 18 ≤ C-MMSE ≤23: mild cognitive impairment; (3) 10 ≤ C-MMSE ≤17: moderate cognitive impairment; and (4) 0 ≤ C-MMSE ≤9: severe cognitive impairment. Cognitive impairment (C-MMSE ≤23) was used as the main outcome variable.

### 2.3. Measurements of cardiovascular risk factors

In the follow-up duration, CVRFs including systolic blood pressure (SBP), diastolic blood pressure (DBP), mean arterial pressure (MAP), pulse pressure (PP), and body mass index (BMI), were continuously measured. After participants were required to rest for 5 min, blood pressure was measured on the right arm at heart level in a sitting position by trained internists using a mercury sphygmomanometer. The blood pressure was continuously measured twice, with at least one-minute interval between the two measurements. According to Korotkoff Phase I and V sound, SBP and DBP values were determined, respectively. MAP was further calculated based on the formula 1/3(SBP) + 2/3(DBP). PP was calculated as (SBP)-(DBP). Body weight (in kilograms) and height (in centimeters) were measured by trained interviewers. BMI (kg/m^2^) was computed by dividing body weight (kg) by the square of body height (m). Changes in CVRFs during the study were calculated as their values at the end of the follow-up minus those at baseline.

### 2.4. Measurements of covariates

In the present study, we selected baseline covariates, such as sociodemographic characteristics, lifestyle, and medical examination, which may confound the relationships between CVRFs and cognitive impairment based on previous studies (Yi, 2008; Yuan et al., 2019; Zhang et al., 2019). Data on sociodemographic characteristics and lifestyle were obtained by questionnaire. The sociodemographic characteristics included gender (male/female), category of residence areas (city/town/rural), living pattern (with family member(s)/alone), education level, and self-reported economic status (very bad/bad/fair/good or very good). The lifestyle included smoking (yes/no), drinking (yes/no), regular exercise (current/past/never), self-reported sleep quality (very bad/bad/fair/good or very good), fresh fruit consumption (rarely or never/ occasionally/ quite often/ almost every day), and vegetable consumption (rarely or never/occasionally/quite often/almost every day). The medical examinations were from self-reported hospital diagnoses, which included hypertension (yes/no), diabetes (yes/no), heart disease (yes/no), cerebrovascular disease (yes/no), and cancer (yes/no).

### 2.5. Statistical analysis

First, categorical variables were presented as numbers (percentages), and continuous variables were expressed as median (interquartile range) because of nonnormal distribution. The differences in the baseline characteristics across different cognitive functions were compared using Chi-square and the Mann–Whitney U test.

Second, in the longitudinal development of CVRFs (SBP, DBP, MAP, PP, and BMI) during the follow-up, the latent growth mixture model (LGMM) was adopted to investigate heterogeneity and identify subgroups of participants who shared similar underlying trajectories of CVRFs. The functional form of the trajectories varied across a number of different orders of polynomials, and the best-fitting polynomial form can be specified for each trajectory separately. This property can depict differential age-related patterns of changes in CVRFs observed over the follow-up period in our samples. The trajectories of changes in CVRFs during the follow-up duration were modeled as follows. Because CVRFs were not normally distributed, the rank-order normalization procedure was first used to generate CVRFs with standard normal distributions. The first step was to determine the most appropriate number of trajectories based on the quadratic model. The number of trajectories was chosen based on better goodness of fit (2*ΔBIC >10, indicating better goodness-of-fit for n-class model than n-1 class model), internal reliability (mean posterior probability >0.65 for each latent class, reflecting an acceptable uncertainty of posterior classification) (Marioni et al., 2014; Norris et al., 2021), clinical plausibility, and interpretability (the size of the smallest class size ≥45 subjects). At last, the maximum 2*ΔBIC between the quadratic and cubic order terms was adopted to determine the shape of each trajectory group. Five trajectory models for SBP, DBP, MAP, PP, and BMI were generated based on adequate fit to data, classification accuracy, and clinical interpretability. All participants were assigned to their subgroups with the highest posterior probability for subsequent analyses.

Third, Cox regression models were used to evaluate the hazard ratios (HRs) of cognitive impairment across different trajectories of CVRFs. The age at which the participants first experienced cognitive impairment was used as the timescale for survival analyses. The participants who never experienced cognitive impairment were considered as censored observations and the censoring time was the age of the last assessment of cognitive function. Four adjusted Cox models were established. Model 1 was the basic model without covariates. Model 2 was established based on Model 1 after adjustments for the sociodemographic characteristics. Model 3 was established based on Model 2 with further adjustments for lifestyle. Model 4 was established based on Model 3 with further adjustments for the medical examination.

Fourth, we repeated Cox regression for participants with different sex, residence areas, and living pattern. The interactions were also tested by comparing models with and without a product term between trajectories of CVRFs and sex/residence areas/living patterns. Additionally, a series of sensitivity analyses were conducted to test the robustness of our findings. First, Cox regression was conducted with moderate/severe cognitive impairment (C-MMSE ≤ 17) as the outcome variable. Second, Cox regression was repeated with severe cognitive impairment (C-MMSE ≤ 9) as the unique outcome. Third, we excluded participants with a history of hypertension, diabetes, heart disease, cerebrovascular disease, and cancer at the baseline because major chronic diseases could influence both CVRF trajectories and cognitive impairment. Fourth, we imputed all missing covariates using multiple imputations to test the influence of missing variables. Fifth, we used restricted cubic splines to express the potentially non-linear relationship between changes in CVRFs and the risk of cognitive impairment. A two-sided *p* < 0.05 was considered statistically significant in all analyses. LGMM analyses were conducted using THE TRAJ procedure in Stata 12.0 (StataCorp LP, College Station, TX, USA). The restricted cubic splines were performed in R (version 4.1.2) with the package “rms.” All other statistical analyses were performed in SPSS 26.0 (IBM SPSS Inc., New York, NY, USA).

## 3. Results

### 3.1. Basic characteristics

This study included 5,164 adults aged ≥60 (median age: 75 years). Among them, 51.8% were male, 35.0% resided in urban areas, and 85.9% lived with their families. During a median 8-year follow-up, the prevalence of cognitive impairment (C-MMSE ≤23) was 40.1% among the participants. There were significant differences in the baseline characteristics across different cognitive functions (Table 1). Compared with the participants without cognitive impairment, participants with cognitive impairment were more likely to be female, rural residents, living alone, less educated, less economically well-off, and eating fewer fruits and vegetables. At the beginning of the follow-up, most CVRFs (e.g., SBP, DBP, and MAP) did not differ in the different cognitive functions, while they differed significantly at the end of the follow-up (Table 2).

**Table 1:** Baseline characteristics of CLHLS participants with different cognitive functions (cognitive impairment as the outcome).

Characteristics	No cognitive impairment (24–30)	Cognitive impairment (0–23)	Overall	*p* value
No. of participants	3,093 (59.9)	2,071 (40.1)	5,164	
Age (years)				<0.001
median (interquartile range)	71.0 (12.0)	81.0 (15.0)	75.0 (16.0)	
Gender (%)				<0.001
Male	1,855 (60.0)	822 (39.7)	2,677 (51.8)	
Female	1,238 (40.0)	1,249 (60.3)	2,487 (48.2)	
Category of residence areas (%)				<0.001
City	568 (18.4)	283 (13.7)	851 (16.5)	
Town	568 (18.4)	388 (18.7)	956 (18.5)	
Rural	1,957 (63.3)	1,400 (67.6)	3,357 (65.0)	
Living pattern (%)				<0.001
Living with family member(s)	2,721 (88.1)	1,708 (82.6)	4,429 (85.9)	
Living alone	367 (11.9)	361 (17.4)	728 (14.1)	
Education level (years)				<0.001
median (interquartile range)	3.0 (6.0)	0.0 (2.0)	1.0 (4.0)	
Self-reported economic status (%)				<0.001
Very poor	50 (1.6)	48 (2.3)	98 (1.9)	
Poor	345 (11.2)	298 (14.4)	643 (12.5)	
Fair	2,149 (69.6)	1,405 (68.0)	3,554 (68.9)	
Rich	499 (16.2)	302 (14.6)	801 (15.5)	
Very rich	45 (1.5)	14 (0.7)	59 (1.1)	
Smoking (%)				<0.001
Yes	963 (31.2)	392 (18.9)	1,355 (26.2)	
No	2,128 (68.8)	1,679 (81.1)	3,807 (73.8)	
Drinking (%)				<0.001
Yes	846 (27.4)	431 (20.8)	1,277 (24.7)	
No	2,243 (72.6)	1,640 (79.2)	3,883 (75.3)	
Regular exercise (%)				<0.001
Current	1,177 (38.1)	639 (30.9)	1816 (35.2)	
Past	200 (6.5)	171 (8.3)	371 (7.2)	
Never	1,708 (55.2)	1,256 (60.6)	2,964 (57.4)	
Self-reported sleep quality (%)				0.014
Very bad	21 (0.7)	17 (0.8)	38 (0.7)	
Bad	291 (9.4)	188 (9.1)	479 (9.3)	
Fair	633 (20.5)	474 (22.9)	1,107 (21.4)	
Good	1,614 (52.2)	1,097 (53.0)	2,711 (52.5)	
Very good	534 (17.3)	295 (14.2)	829 (16.1)	
Fresh fruit consumption (%)				<0.001
rarely or never	558 (18.0)	475 (22.9)	1,033 (20.0)	
occasionally	1,111 (35.9)	863 (41.7)	1974 (38.2)	
quite often	987 (31.9)	547 (26.4)	1,534 (29.7)	
almost everyday	437 (14.1)	186 (9.0)	623 (12.1)	
Fresh vegetable consumption (%)				<0.001
rarely or never	31 (1.0)	31 (1.5)	62 (1.2)	
occasionally	207 (6.7)	178 (8.6)	385 (7.5)	
quite often	928 (30.1)	713 (34.4)	1,641 (31.8)	
almost every day	1922 (62.2)	1,149 (55.5)	3,071 (59.5)	
Hypertension (%)				0.007
Yes	854 (28.0)	502 (24.6)	1,356 (26.6)	
No	2,196 (72.0)	1,540 (75.4)	3,736 (73.4)	
Diabetes (%)				0.002
Yes	242 (7.9)	116 (5.7)	358 (7.0)	
No	2,812 (92.1)	1,923 (94.3)	4,735 (93.0)	
Heart disease (%)				0.006
Yes	415 (13.6)	225 (11.0)	640 (12.5)	
No	2,638 (86.4)	1,822 (89.0)	4,460 (87.5)	
Cerebrovascular disease (%)				0.555
Yes	207 (6.8)	148 (7.2)	355 (7.0)	
No	2,840 (93.2)	1,901 (92.8)	4,741 (93.0)	
Cancer (%)				0.019
Yes	64 (2.1)	25 (1.2)	89 (1.8)	
No	2,962 (97.9)	2,007 (98.8)	4,969 (98.2)	

Data are expressed as numbers (percentages) or median (interquartile range).

**Table 2:** CVRFs among participants with and without cognitive impairment.

CVRFs	No cognitive impairment (24–30)	Cognitive impairment (0–23)	*p* value
No. of participants	3,093 (59.9)	2,071 (40.1)	
Beginning of the follow-up
SBP (mmHg)	134.81 (20.06)	133.82 (19.87)	0.084
DBP (mmHg)	81.92 (11.41)	82.04 (11.45)	0.701
BMI (kg/m2)	21.38 (3.77)	20.03 (3.72)	<0.001
MAP (mmHg)	99.56 (12.16)	99.27 (12.15)	0.413
PP (mmHg)	52.93 (17.98)	51.69 (17.56)	0.016
End of the follow-up
SBP (mmHg)	140.17 (21.05)	138.80 (22.10)	0.028
DBP (mmHg)	80.54 (11.41)	79.46 (12.07)	0.002
BMI (kg/m2)	22.40 (4.01)	20.85 (4.00)	<0.001
MAP (mmHg)	100.39 (12.84)	99.18 (13.38)	0.001
PP (mmHg)	59.55 (17.52)	59.16 (18.80)	0.464
Changes in CVRFs
SBP (mmHg)	5.41 (26.62)	5.03 (27.76)	0.636
DBP (mmHg)	−1.36 (15.10)	−2.60 (15.76)	0.006
BMI (kg/m^2^)	1.01 (3.97)	0.81 (4.35)	0.094
MAP (mmHg)	0.86 (16.22)	−0.10 (16.70)	0.045
PP (mmHg)	6.66 (23.48)	7.49 (24.93)	0.240

Data are expressed as mean (standard deviation). Changes in CVRFs during the study was calculated as their values at the end of the follow-up minus those at baseline. SBP, systolic blood pressure; DBP, diastolic blood pressure; BMI, body mass index; MAP, mean arterial pressure; PP, pulse pressure.

Meanwhile, moderate/severe cognitive impairments (C-MMSE ≤17) were developed in 21.4% of the participants, and severe cognitive impairment (C-MMSE ≤9) were observed in 11.6% of the participants (Supplementary Tables S1, S2). Supplementary Table S3 shows that patients excluded from the analysis had very different characteristics from those included, including age and the prevalence of heart disease and diabetes, among others. The included patients were younger, more frequently male, and more likely to be smokers but less likely to have diabetes, heart disease, and cerebrovascular disease.

### 3.2. Trajectories of changes in CVRFs during the follow-up

In trajectory modeling, the number of trajectories was determined by using a quadratic model, and all fit indices are shown in Supplementary Tables S4–S8. The shape of each trajectory group was determined by comparing the goodness of fit (2*ΔBIC > 10) between quadratic and cubic order terms, as shown in Supplementary Tables S9–S13. SBP was taken as an example to illustrate the determination of the optimal model. In the first step, the indicator 2*ΔBIC > 10 indicated that the goodness of fit is better for the n-class model than the *n*−1 class model. Also, the proportion of subjects classified in each group with a posterior probability >0.65 and a minimum class sample size was greater than 45 in the four-class model. Considering all metrics, clinical plausibility, and interpretability, the four-class model was chosen as the optimal model for the SBP trajectory group. The optimal SBP trajectory model was determined after assessing the goodness of fit (maximum 2*ΔBIC) for the four-trajectory solution shapes (Supplementary Table S14). Based on the same indicators and principles, the optimal trajectory models for DBP, MAP, PP, and BMI were also determined (Supplementary Table S14). Supplementary Table S15 presents the parameter estimation results of modeling the trajectories of CVRFs.

**Figure 1:**
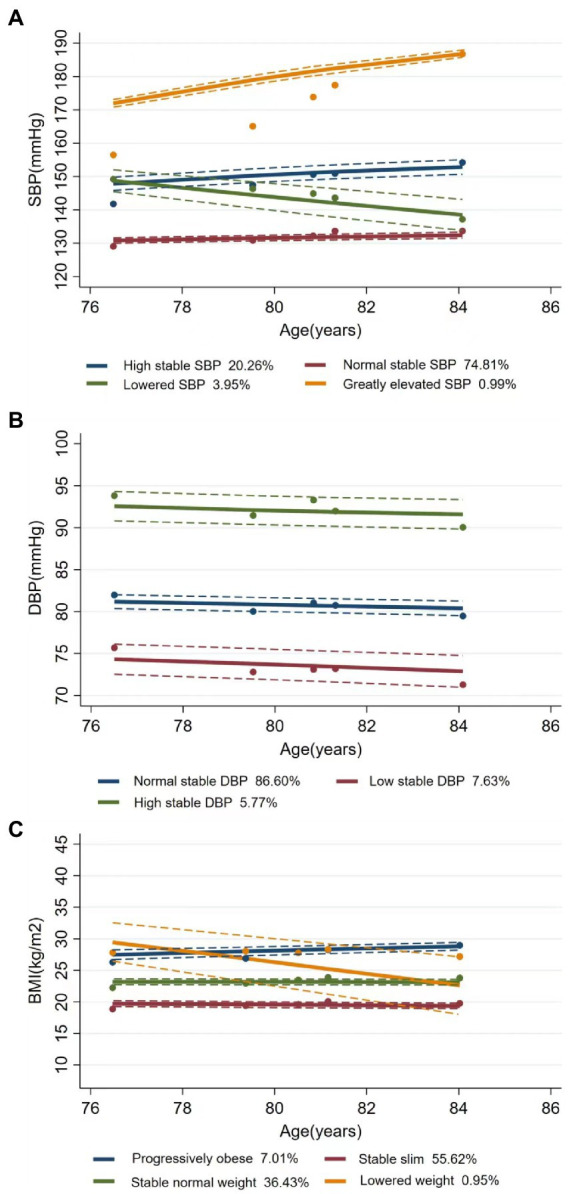
Latent trajectories of SBP **(A)**, DBP **(B)**, and BMI **(C)** for Chinese older people. Estimated trajectories (solid lines), observed group means for each survey (dot symbols). Dashed lines are approximated 95% pointwise CIs on the estimated trajectories. SBP, systolic blood pressure; DBP, diastolic blood pressure; BMI, body mass index.

Figure 1 shows the trends and trajectories of changes in CVRFs, including SBP, DBP, and BMI. During the follow-up visits, SBP trajectory was classified into four classes: (1) high stable SBP (*n* = 1,046, 20.26%) with consistently high SBP level at about 150 mmHg, (2) normal stable SBP (*n* = 3,863, 74.81%) with the stable SBP at about 130 mmHg, (3) lowered SBP (*n* = 204, 3.95%) with decreasing SBP from 149 mmHg to 137 mmHg, and (4) greatly elevated SBP (*n* = 51, 0.99%) with a large increase in SBP from 157 mmHg to 188 mmHg. The three-class DBP trajectory was obtained as follows: (1) normal stable DBP (*n* = 4,472, 86.60%) with a maintained normal stable DBP at about 81 mmHg, (2) low stable DBP (*n* = 394, 7.63%) with consistently low DBP around 74 mmHg, and (3) high stable DBP (*n* = 298, 5.77%) with consistently high DBP at about 92 mmHg. For the four-class BMI trajectory, 7.01% (*n* = 362) of the participants were progressively obese (from 26 kg/m^2^ to 29 kg/m^2^), 55.62% (*n* = 2,872) were stably slim, 36.43% (*n* = 1,881) maintained a stable normal weight of about 23 kg/m^2^, and only 0.95% (*n* = 49) had declined weight from 29 to 27 kg/m^2^. MAP was classified as (1) low stable MAP (*n* = 483, 9.35%), (2) normal stable MAP (*n* = 4,196, 81.25%), and (3) high stable MAP (*n* = 485, 9.39%) (Supplementary Figure S2). PP included (1) elevated PP (*n* = 385, 7.46%), (2) normal stable PP (*n* = 4,668, 90.40%), and (3) lowered PP (*n* = 111, 2.15%) (Supplementary Figure S3).

### 3.3. Effects of CVRF trajectories on the risk of cognitive impairment

**Figure 2:**
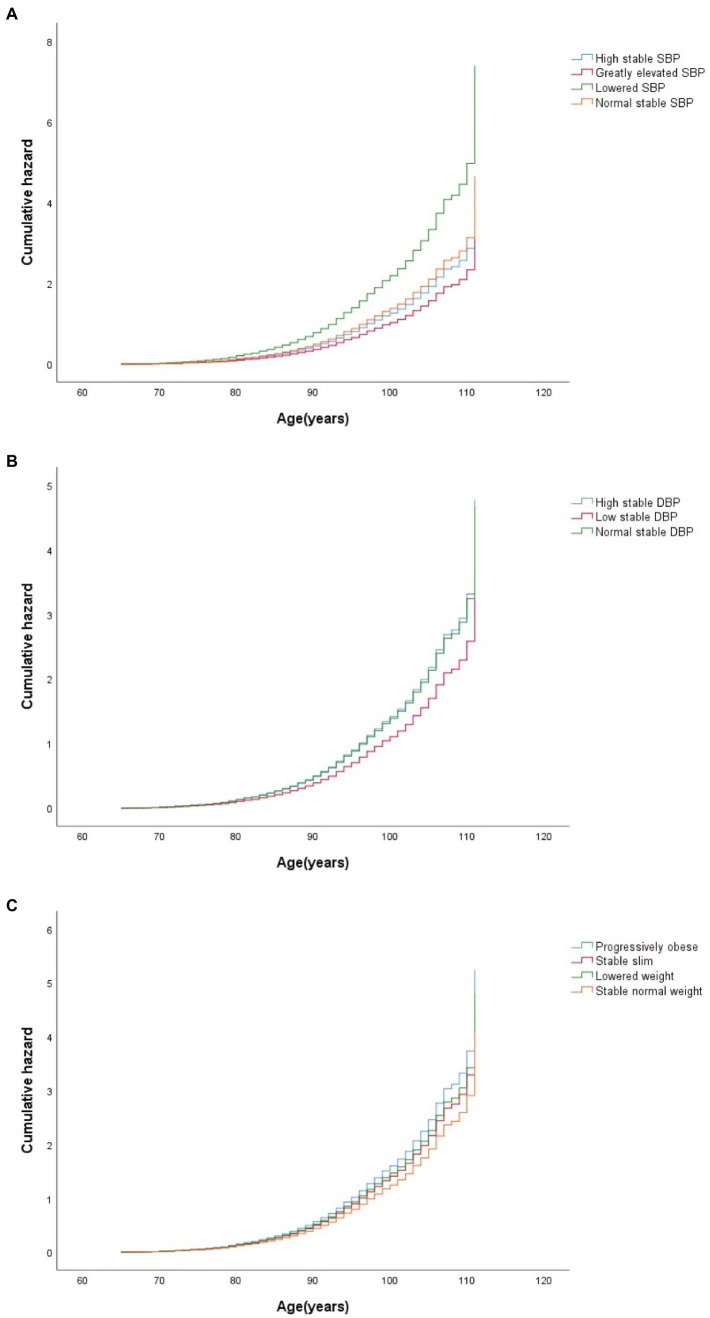
Curves of the cumulative hazard function of cognitive impairment by trajectory classes of SBP **(A)**, DBP **(B)**, and BMI **(C)** in the final adjusted model. SBP, systolic blood pressure; DBP, diastolic blood pressure; BMI, body mass index.

The hazard ratios (HRs) of cognitive impairment by different CVRF trajectories using Cox regression are presented in Table 3. With the normal stable SBP group as a reference, a higher risk of cognitive impairment was observed in the lowered SBP group [adjusted HR (aHR) = 1.67; 95% CI: 1.24–2.25] in a univariate model. After adjusting for different covariates, including sociodemographic characteristics, lifestyle, and physical examination, sequentially from Model 2 to Model 4, the lowered SBP group had a higher risk of cognitive impairment (aHR = 1.59; 95% CI: 1.17–2.16) compared to the normal stable SBP group, and the low stable DBP group had a lower risk of cognitive impairment (aHR = 0.80; 95% CI: 0.67–0.95) compared to the normal stable DBP group in a univariate model. After additional adjustments for covariates, a lower risk of cognitive impairment was still observed in the low stable DBP group than in the normal stable DBP group (aHR = 0.80; 95% CI: 0.66–0.96). Compared with the group with stable normal weight, both the progressively obese group and the stable slim group had a higher risk for cognitive impairment in Model 4 (aHR = 1.28, 95%CI: 1.02–0.62 for the former and aHR = 1.13, 95%CI: 1.02 –1.25 for the latter). Figure 2 shows the adjusted cumulative hazard function curves of cognitive impairment by SBP, DBP, and BMI trajectories, consistent with the main results.

**Table 3:** Effects of CVRF trajectories on the risk of cognitive impairment.

Variables	Model 1	Model 2	Model 3	Model 4
Normal stable SBP as reference	
High stable SBP	0.94 (0.84, 1.06)	0.92 (0.82, 1.04)	0.94 (0.83, 1.05)	0.92 (0.81, 1.03)
Greatly elevated SBP	0.82 (0.51, 1.32)	0.74 (0.46, 1.19)	0.73 (0.45, 1.18)	0.75 (0.46, 1.21)
Lowered SBP	1.67 (1.24, 2.25)*	1.68 (1.24, 2.27)*	1.66 (1.23, 2.25)*	1.59 (1.17, 2.16)*
Normal stable DBP as reference	
High stable DBP	0.98 (0.80, 1.19)	0.99 (0.81, 1.20)	1.00 (0.82, 1.21)	1.02 (0.84, 1.25)
Low stable DBP	0.80 (0.67, 0.95)*	0.83 (0.70, 0.99)*	0.81 (0.68, 0.97)*	0.80 (0.66, 0.96)*
Stable normal weight as reference	
Progressively obese	1.255 (1.003, 1.570)*	1.28 (1.02, 1.61)*	1.30 (1.04, 1.64)*	1.28 (1.02, 1.62)*
Stable slim	1.25 (1.14, 1.38)*	1.14 (1.04, 1.26)*	1.12 (1.02, 1.24)*	1.13 (1.02, 1.25)*
Lowered weight	1.46 (0.82, 2.58)	1.30 (0.73, 2.32)	1.30 (0.73, 2.31)	1.18 (0.66, 2.11)
Normal stable MAP as reference	
Low stable MAP	1.01 (0.87, 1.17)	1.03 (0.88, 1.20)	1.02 (0.87, 1.18)	1.01 (0.86, 1.18)
High stable MAP	1.07 (0.91, 1.24)	1.02 (0.87, 1.19)	1.03 (0.88, 1.20)	1.02 (0.87, 1.20)
Normal stable PP as reference	
Elevated PP	0.78 (0.65, 0.93)*	0.76 (0.64, 0.92)*	0.77 (0.64, 0.93)*	0.76 (0.63, 0.92)*
Lowered PP	2.58 (1.65, 4.04)*	2.79 (1.78, 4.36)*	2.75 (1.75, 4.30)*	2.64 (1.66, 4.19)*

SBP, systolic blood pressure. DBP, diastolic blood pressure. MAP, mean arterial pressure. PP, pulse pressure. Hazard ratios (95% confidence intervals) are presented. Model 1 was adjusted for no covariates. Model 2 was adjusted for gender, category of residence areas, living pattern, education level, and self-reported economic status based on model 1. Model 3 was adjusted for model 2 plus smoking, drinking, regular exercise, self-reported sleep quality, fresh fruit consumption, and vegetable consumption. Model 4 was adjusted for model 3 plus hypertension, diabetes, heart disease, cerebrovascular disease, and cancer. *p < 0.05.

Compared to the participants with normal stable PP, the participants with lowered PP were more likely to suffer from cognitive impairment (aHR = 2.64; 95% CI: 1.66–4.19 in Model 4), while the participants with elevated PP had a lower risk of cognitive impairment (aHR = 0.76; 95% CI: 0.63–0.92 in Model 4). However, there was no statistically significant association between the MAP trajectory groups and the risk of cognitive impairment. The adjusted cumulative hazard of cognitive impairment by MAP and PP trajectories, also show consistent results (Supplementary Figures S4, S5).

### 3.4. Subgroup analyses

According to the baseline characteristics, such as gender, residence areas, and living pattern, the associations between CVRF trajectories and cognitive impairment across different subgroups were broadly consistent with our main findings. Across subgroups, the positive association between the lowered SBP group and the risk for cognitive impairment remained significant (Supplementary Table 16), and the effect of living pattern on appreciable modification of SBP was significant (interaction value of *p* < 0.05). Low stable DBP was associated with a lower risk of cognitive impairment only in men (aHR = 0.63, 95%CI: 0.47–0.85), and only the effect of gender on appreciable modification of DBP was significant (Supplementary Table 17). The associations of trajectories of other CVRFs with cognitive impairment were unchanged after stratification by gender, residence areas, and living pattern, showing no statistically significant interactions (Supplementary Tables S18–S20).

### 3.5. Sensitivity analyses

When moderate/severe cognitive impairment (C-MMSE ≤ 17) and severe cognitive impairment (C-MMSE ≤ 9), were separately used as the outcome variables (Supplementary Tables S21, S22), the results were similar to the main findings. The results remained consistent in general after excluding subjects with a history of hypertension, diabetes, heart disease, cerebrovascular disease and cancer at the baseline (Supplementary Table S23). After imputing missing values of all covariates using multiple imputations, all results remained the same (Supplementary Table S23). The restricted cubic splines were further adopted to explore the relationships between changes in CVRFs during the life course and risk of cognitive impairment after adjustment for possible confounders (Supplementary Figures S6–S10). The more amount of reduction in SBP contributed to a higher hazard of cognitive impairment. The increments in SBP ranging from 5 to 42 mmHg, decreased the risk of cognitive impairment. However, the associations of changes in SBP with risk of cognitive impairment did not reach the predetermined level of statistical significance. More DBP lowering and ≥40 mmHg growth in DBP lead to an increasing hazard of cognitive impairment. When PP increased by 7–25 mmHg, the risk of cognitive impairment reduced, and when PP increased by over 25 mmHg, the hazard of cognitive impairment rose. The risk for cognitive impairment grew with the reduction in MAP during the study. The declines in BMI were associated with a higher likelihood of cognitive impairment.

## 4. Discussion

In this nationwide cohort of elderly in China, the developmental trajectories of CVRFs over time were identified using LGMM, and their relationships with cognitive impairment were further explored. The results indicated that the lowered SBP, lowered PP, progressively obese, and stable slim were associated with a higher risk of cognitive impairment, and the low stable DBP and elevated PP were associated with a lower risk of cognitive impairment. The associations were not modified by sociodemographic characteristics, lifestyle, and medical examination, and sensitivity analysis was also conducted to demonstrate the robustness of the results.

In this cohort, the incidence density of cognitive impairment (C-MMSE ≤ 23) in the participants over 60 years was 50.2 per 1,000 person-years. Also, the incidence densities of moderate/severe cognitive impairment (C-MMSE ≤ 17) and severe cognitive impairment (C-MMSE ≤ 9) were 26.7 and 14.5 per 1,000 person-years, respectively. The estimates of the incidence of cognitive impairment varied considerably by country. For example, a 4.7-year follow-up study in the United States showed that the incidence density of mild cognitive impairment was 51 per 1,000 person-years among people over 65 years (Manly et al., 2008). Another study in Italy investigating mild cognitive impairment among participants over 65 years reported an incidence density of 76.8 per 1,000 person-years during a 4-year follow-up period. In contrast, the observed prevalence of cognitive impairment in our study population approached the developed-country level. Because cognitive impairment represents an intermediate stage between normal aging and dementia, recognition of cognitive impairment plays an important role in early intervention and prevention of dementia (Eshkoor et al., 2015). As the population ages, more and more elder people will suffer from cognitive impairment, especially in China. This makes identifying people at risk of developing dementia at an early stage important public health implications.

Four and three unique trajectories were determined for BP and PP, respectively. The percentages of groups with normal stable BP and PP were over 70%, indicating that the vast majority of elderly in our study had little change in blood pressure. Because our subjects were older and had fewer deleterious BP changes compared with the general population, our study had a higher proportion of people with normal BP than in previous studies (Smitson et al., 2017). Similar to our study, a cohort study of the elderly with a median age of 77 years conducted in the United States identified trajectories of increased, little changed, and declined SBP by hierarchical cluster analysis (Smitson et al., 2017). Cheng et al. (2021) also demonstrated four unique BP/PP trajectories: normal, stabilized, elevated, and persistently high BP/PP in the Chinese elderly. In previous studies, the differences in the baseline characteristics and risk of heart disease and cerebrovascular disease among BP/PP trajectory groups were similar to our study (Smitson et al., 2017; Cheng et al., 2021). Thus, the occurrence of lowered BP in our study should not be considered a complication of heart disease and cerebrovascular diseases. Further epidemiological studies will be needed to examine the specific mechanisms that generate these abnormal trajectories. Hakala et al. (2021) also identified four trajectory groups of BMI among participants between young adulthood and midlife based on a 31-year cohort study, consistent with our findings. In general, our results are consistent with previous findings and represent real dynamic trajectories of CVRFs. This suggests heterogeneity in the trajectories of CVRFs in older adults and that subpopulations respond similarly to repeated measures of CVRFs over time.

Our study indicated that lowered SBP change was detrimental to cognitive function and increased the incidence of cognitive impairment by 59% among the elderly. Similarly, a 32-year follow-up study of 1,890 middle-aged and elderly Japanese-American men also demonstrated that people with dementia had a faster decline in SBP compared to those without dementia (Stewart et al., 2009). In Sweden, a 37-year follow-up study showed that among female participants with a mean age of 45 years at the baseline, those with dementia had a greater decline in SBP later in life than those without (Joas et al., 2012). Our study observed that low stable DBP (around 74 mmHg) was associated with a lower risk of cognitive impairment, but excessive reduction of DBP increased its risk. Similar to our findings, a cross-sectional study showed that those with a mean DBP of 77 mmHg had the highest cognitive function scores among those 65 years and older (Morris et al., 2002). In a 9-year longitudinal study, Glynn et al. (1999) found a U-shaped association between cognitive impairment and DBP and demonstrated that lower DBP (<70 mm Hg) and higher DBP (≥80 mm Hg) were associated with an increased risk of cognitive impairment. Gao et al. (2021) also found that persistently low stable rather than significantly reduced DBP might be protective against cognitive impairment. Furthermore, our results suggested that lowered PP was associated with an increased risk of cognitive impairment. Molander et al. (2010) also confirmed a larger decrease in PP in dementia cases, consistent with our findings. However, not entirely consistent with previous findings (Li et al., 2022), our results showed that elevated PP was a protective factor against cognitive impairment, but ≥25 mmHg growth in PP increased the risk of cognitive impairment. The possible mechanisms of blood pressure worsening cognitive function include promoting disturbances in amyloid clearance, reduced cerebral perfusion, and white matter damage (Bink et al., 2013; Wanleenuwat et al., 2019). The findings suggest that it is feasible to identify people who are more likely to develop cognitive impairment based on changes in blood pressure and are of great significance to improving cognitive function, delaying the onset of dementia, and reducing family and social burdens.

In our study, stable slim and progressively obese were observed to increase the risk of cognitive impairment. Similar to our study, two cohort studies on the Chinese elderly showed that significant weight loss or being underweight might be significant risk factors for cognitive impairment (Ren et al., 2021; Wu et al., 2021). However, the evidence for the relationship between obesity and cognitive impairment is conflicting, with obesity-related favorable and detrimental factors conjointly determining cognitive outcomes. Obesity is thought to affect hypertension, type 2 diabetes, and cardiovascular disease, thereby increasing the risk of dementia (Luchsinger and Gustafson, 2009) consistent with our findings. However, according to clinical data from the U.S. National Alzheimer Coordinating Center, a high BMI is associated with slower progression of amnestic mild cognitive impairment (Besser et al., 2014). Perhaps, this can be explained by the neuroprotective effect of leptin, which rises with obesity (Lieb et al., 2009). More research is still needed to elucidate this association.

The strength of our study is the use of a large, exceptional cohort, which provides higher statistical power. Second, the prospective cohort study design allowed us to obtain complete data on changes in CVRFs and analyze confirmed cognitive impairment. Third, considering CVRFs are not static but change with age over the course of life, we used LGMM to model the trajectory of CVRFs over time and explored the relationships between long-term trajectories of CVRFs and cognitive impairment. Our study fills certain knowledge gaps in the relationships between long-term trends in CVRFs and cognitive function among the Chinese elderly. Fourth, our study detects the associations between CVRF trajectory and various degrees of cognitive impairment. Meanwhile, the robustness of our findings was further evaluated using a series of sensitivity analyses.

Admittedly, some limitations of our study should be noted when interpreting the results. First, we adjusted for the key personal characteristics and lifestyle behaviors in the analyses, but other unmeasured confounders, such as antihypertensive drugs and plasma glucose, may still influence our results. Second, the information on lifestyle behaviors was self-reported by participants at the baseline. Therefore, we could not rule out the possibility of information bias. Third, the lifestyle behaviors were assessed at the baseline and not updated during the follow-up because there might be a reverse causal relationship between lifestyle changes and cognitive impairment as the population ages. Fourth, our estimates are based on observational data and do not imply certain causality. Fifth, although CLHLS is a nationally representative sample, we excluded more than 20,000 participants and only included 5,164 adults who had at least three visits during the follow-up duration. However, the excluded participants had different baseline characteristics from those included, which might indicate selection bias. Sixth, we did not investigate whether the participants developed dementia during the follow-up, nor could we explore the relationship between CVRFs and dementia. Seventh, the derived classes from LGMM were used in another program for survival analysis, and potential posterior bias was inevitable.

## 5. Conclusion

Our study used LGMM to describe the direction and magnitude of changes in CVRFs over time. SBP and BMI were divided into four trajectory subgroups, and DBP, MAP, and PP were classified into three trajectory subgroups. The results showed that lowered SBP and PP in the elderly were associated with an increased risk of cognitive impairment. Although low stable DBP decreased the risk of cognitive impairment, more DBP lowering would lead to the occurrence of cognitive impairment. The elevated PP decreased the hazard of the cognitive impairment, but ≥25 mmHg growth in PP contributed to a higher risk of cognitive impairment. Participants with progressively obese and stable slim were more likely to suffer from cognitive impairment. The study has important implications in preventing the occurrence of cognitive impairment by controlling SBP, DBP, BMI, and PP of the elderly.

## Data availability statement

Publicly available datasets were analyzed in this study. These data can be found here: https://opendata.pku.edu.cn/dataverse/CHADS.

## Ethics statement

The studies involving human participants were reviewed and approved by Research Ethics Committees of Duke University and Peking University (IRB00001052-13074). The patients/participants provided their written informed consent to participate in this study.

## Author contributions

LP and XD conceived the study and drafted the manuscript. XD, YD, CK, PR, MY, SZ, and JC contributed to data acquisition, analyses, and interpretation. XD, LP, YZ, FC, JZ, and DW participated in the critical revision of the manuscript for important intellectual content. All authors contributed to the critical revisions and approved the final version of the manuscript.

## Funding

This work was supported by the National Natural Science Foundation of China (Grant Nos. 72174167 and 81602928), the Natural Science Foundation of Shaanxi (Grant No. 2021JM-031), and the National Key R&D Program of China (2017YFC0907200, 2017YFC0907201).

## Conflict of interest

The authors declare that the research was conducted without any commercial or financial relationships that could be construed as potential conflicts of interest.

## Publisher’s note

All claims expressed in this article are solely those of the authors and do not necessarily represent those of their affiliated organizations, or those of the publisher, the editors and the reviewers. Any product that may be evaluated in this article, or claim that may be made by its manufacturer, is not guaranteed or endorsed by the publisher.
