# Supplementary material for: Associations between trajectories of cardiovascular risk factor change and cognitive impairment in Chinese elderly: A nationwide cohort study
Source: Front Aging Neurosci. 2023 Feb 10;15:1084136. doi: 10.3389/fnagi.2023.1084136 (PMC9950264; doi:10.3389/fnagi.2023.1084136)
Supplement: Supplementary file 1 [file Table_1.docx]

**Supplementary Table 1 Baseline characteristics of CLHLS participants with different cognitive function (Moderate/severe cognitive impairment as the outcome).**

| Characteristics | no and mild cognitive impairment (18−30) | moderate and severe cognitive impairment (0−17) | Overall | P value |
| --- | --- | --- | --- | --- |
| **No. of participants** | 4060 (78.6) | 1104 (21.4) | 5164 |  |
| **Age (years)** |  |  |  | <0.001 |
| median (interquartile range) | 72.0 (14.0) | 84.0 (13.0) | 75.0 (16.0) |  |
| **Gender (%)** |  |  |  | <0.001 |
| Male | 2262 (55.7) | 415 (37.6) | 2677 (51.8) |  |
| Female | 1798 (44.3) | 689 (62.4) | 2487 (48.2) |  |
| **Category of residence areas (%)** |  |  |  | 0.119 |
| City | 684 (16.8) | 167 (15.1) | 851 (16.5) |  |
| Town | 731 (18.0) | 225 (20.4) | 956 (18.5) |  |
| Rural | 2645 (65.1) | 712 (64.5) | 3357 (65.0) |  |
| **Living pattern (%)** |  |  |  | 0.001 |
| Living with family member(s) | 3516 (86.7) | 913 (82.8) | 4429 (85.9) |  |
| Living alone | 539 (13.3) | 189 (17.2) | 728 (14.1) |  |
| **Education (years)** |  |  |  | <0.001 |
| median (interquartile range) | 2.0 (6.0) | 0.0 (2.0) | 1.0 (5.0) |  |
| **Self-reported economic status (%)** |  |  |  | 0.023 |
| Very poor | 66 (1.6) | 32 (2.9) | 98 (1.9) |  |
| Poor | 478 (11.8) | 165 (15.0) | 643 (12.5) |  |
| Fair | 2835 (69.9) | 719 (65.2) | 3554 (68.9) |  |
| Rich | 625 (15.4) | 176 (16.0) | 801 (15.5) |  |
| Very rich | 49 (1.2) | 10 (0.9) | 59 (1.1) |  |
| **Smoking (%)** |  |  |  | <0.001 |
| Yes | 1163 (28.7) | 192 (17.4) | 1355 (26.2) |  |
| No | 2895 (71.3) | 912 (82.6) | 3807 (73.8) |  |
| **Drinking (%)** |  |  |  | <0.001 |
| Yes | 1064 (26.2) | 213 (19.3) | 1277 (24.7) |  |
| No | 2992 (73.8) | 891 (80.7) | 3883 (75.3) |  |
| **Regular exercise (%)** |  |  |  | 0.008 |
| Current | 1479 (36.4) | 337 (30.5) | 1816 (35.2) |  |
| Past | 268 (6.6) | 103 (9.3) | 371 (7.2) |  |
| Never | 2303 (56.7) | 661 (59.9) | 2964 (57.4) |  |
| **Self-reported sleep quality (%)** |  |  |  | 0.310 |
| Very bad | 28 (0.7) | 10 (0.9) | 38 (0.7) |  |
| Bad | 381 (9.4) | 98 (8.9) | 479 (9.3) |  |
| Fair | 856 (21.1) | 251 (22.7) | 1107 (21.4) |  |
| Good | 2132 (52.5) | 579 (52.4) | 2711 (52.5) |  |
| Very good | 663 (16.3) | 166 (15.0) | 829 (16.1) |  |
| **Fresh fruit consumption (%)** |  |  |  | <0.001 |
| rarely or never | 794 (19.6) | 239 (21.6) | 1033 (20.0) |  |
| occasionally | 1500 (36.9) | 474 (42.9) | 1974 (38.2) |  |
| quite often | 1247 (30.7) | 287 (26.0) | 1534 (29.7) |  |
| almost everyday | 519 (12.8) | 104 (9.4) | 623 (12.1) |  |
| **Fresh vegetable consumption (%)** |  |  |  | 0.004 |
| rarely or never | 48 (1.2) | 14 (1.3) | 62 (1.2) |  |
| occasionally | 290 (7.2) | 95 (8.6) | 385 (7.5) |  |
| quite often | 1262 (31.1) | 379 (34.3) | 1641 (31.8) |  |
| almost everyday | 2455 (60.5) | 616 (55.8) | 3071 (59.5) |  |
| **Hypertension (%)** |  |  |  | 0.002 |
| Yes | 1106 (27.6) | 250 (23.0) | 1356 (26.6) |  |
| No | 2897 (72.4) | 839 (77.0) | 3736 (73.4) |  |
| **Diabetes (%)** |  |  |  | 0.001 |
| Yes | 306 (7.6) | 52 (4.8) | 358 (7.0) |  |
| No | 3700 (92.4) | 1035 (95.2) | 4735 (93.0) |  |
| **Heart disease (%)** |  |  |  | 0.104 |
| Yes | 519 (12.9) | 121 (11.1) | 640 (12.5) |  |
| No | 3491 (87.1) | 969 (88.9) | 4460 (87.5) |  |
| **Cerebrovascular disease (%)** |  |  |  | 0.368 |
| Yes | 272 (6.8) | 83 (7.6) | 355 (7.0) |  |
| No | 3729 (93.2) | 1012 (92.4) | 4741 (93.0) |  |
| **Cancer (%)** |  |  |  | 0.066 |
| Yes | 77 (1.9) | 12 (1.1) | 89 (1.8) |  |
| No | 3899 (98.1) | 1070 (98.9) | 4969 (98.2) |  |

Data are expressed as numbers (percentages) or median (interquartile range).

**Supplementary Table 2 Baseline characteristics of CLHLS participants with different cognitive function. (Severe cognitive impairment as the outcome).**

| Characteristics | no, mild and moderate cognitive impairment (10−30) | severe cognitive impairment (0−9) | Overall | P value |
| --- | --- | --- | --- | --- |
| **No. of participants** | 4564 (88.4) | 600 (11.6) | 5164 |  |
| **Age (years)** |  |  |  | <0.001 |
| median (interquartile range) | 73.0 (15.0) | 85.0 (12.0) | 75.0 (16.0) |  |
| **Gender (%)** |  |  |  | <0.001 |
| Male | 2442 (53.5) | 235 (39.2) | 2677 (51.8) |  |
| Female | 2122 (46.5) | 365 (60.8) | 2487 (48.2) |  |
| **Category of residence areas (%)** |  |  |  | 0.084 |
| City | 738 (16.2) | 113 (18.8) | 851 (16.5) |  |
| Town | 835 (18.3) | 121 (20.2) | 956 (18.5) |  |
| Rural | 2991 (65.5) | 366 (61.0) | 3357 (65.0) |  |
| **Living pattern (%)** |  |  |  | 0.239 |
| Living with family member(s) | 3924 (86.1) | 505 (84.3) | 4429 (85.9) |  |
| Living alone | 634 (13.9) | 94 (15.7) | 728 (14.1) |  |
| **Education (years)** |  |  |  | <0.001 |
| median (interquartile range) | 2.0 (6.0) | 0.0 (3.0) | 1.0 (5.0) |  |
| **Self-reported economic status (%)** |  |  |  | 0.956 |
| Very poor | 81 (1.8) | 17 (2.8) | 98 (1.9) |  |
| Poor | 561 (12.3) | 82 (13.7) | 643 (12.5) |  |
| Fair | 3168 (69.5) | 386 (64.4) | 3554 (68.9) |  |
| Rich | 694 (15.2) | 107 (17.9) | 801 (15.5) |  |
| Very rich | 52 (1.1) | 7 (1.2) | 59 (1.1) |  |
| **Smoking (%)** |  |  |  | <0.001 |
| Yes | 1253 (27.5) | 102 (17.0) | 1355 (26.2) |  |
| No | 3309 (72.5) | 498 (83.0) | 3807 (73.8) |  |
| **Drinking (%)** |  |  |  | 0.014 |
| Yes | 1153 (25.3) | 124 (20.7) | 1277 (24.7) |  |
| No | 3407 (74.7) | 476 (79.3) | 3883 (75.3) |  |
| **Regular exercise (%)** |  |  |  | 0.507 |
| Current | 1625 (35.6) | 191 (31.8) | 1816 (35.2) |  |
| Past | 307 (6.7) | 64 (10.7) | 371 (7.2) |  |
| Never | 2621 (57.4) | 343 (57.2) | 2964 (57.4) |  |
| **Self-reported sleep quality (%)** |  |  |  | 0.402 |
| Very bad | 31 (0.7) | 7 (1.2) | 38 (0.7) |  |
| Bad | 428 (9.4) | 51 (8.5) | 479 (9.3) |  |
| Fair | 966 (21.2) | 141 (23.5) | 1107 (21.4) |  |
| Good | 2402 (52.6) | 309 (51.5) | 2711 (52.5) |  |
| Very good | 737 (16.1) | 92 (15.3) | 829 (16.1) |  |
| **Fresh fruit consumption (%)** |  |  |  | 0.001 |
| rarely or never | 902 (19.8) | 131 (21.8) | 1033 (20.0) |  |
| occasionally | 1712 (37.5) | 262 (43.7) | 1974 (38.2) |  |
| quite often | 1388 (30.4) | 146 (24.3) | 1534 (29.7) |  |
| almost everyday | 562 (12.3) | 61 (10.2) | 623 (12.1) |  |
| **Fresh vegetable consumption (%)** |  |  |  | 0.801 |
| rarely or never | 53 (1.2) | 9 (1.5) | 62 (1.2) |  |
| occasionally | 340 (7.5) | 45 (7.5) | 385 (7.5) |  |
| quite often | 1450 (31.8) | 191 (31.8) | 1641 (31.8) |  |
| almost everyday | 2716 (59.6) | 355 (59.2) | 3071 (59.5) |  |
| **Hypertension (%)** |  |  |  | 0.009 |
| Yes | 1225 (27.2) | 131 (22.2) | 1356 (26.6) |  |
| No | 3276 (72.8) | 460 (77.8) | 3736 (73.4) |  |
| **Diabetes (%)** |  |  |  | 0.189 |
| Yes | 324 (7.2) | 34 (5.7) | 358 (7.0) |  |
| No | 4176 (92.8) | 559 (94.3) | 4735 (93.0) |  |
| **Heart disease (%)** |  |  |  | 0.247 |
| Yes | 574 (12.7) | 66 (11.1) | 640 (12.5) |  |
| No | 3930 (87.3) | 530 (88.9) | 4460 (87.5) |  |
| **Cerebrovascular disease (%)** |  |  |  | 0.049 |
| Yes | 302 (6.7) | 53 (8.9) | 355 (7.0) |  |
| No | 4198 (93.3) | 543 (91.1) | 4741 (93.0) |  |
| **Cancer (%)** |  |  |  | 0.434 |
| Yes | 81 (1.8) | 8 (1.4) | 89 (1.8) |  |
| No | 4389 (98.2) | 580 (98.6) | 4969 (98.2) |  |

Data are expressed as numbers (percentages) or median (interquartile range).

**Supplementary Table 3 Baseline characteristics of participants excluded and included in the analysis.**

| Characteristics | Excluded in the analysis  (N=21313) | Included in the analysis  (N=5164) | P-value |
| --- | --- | --- | --- |
| **Age (years)** |  |  | <0.001 |
| median (interquartile range) | 91.0 (17.0) | 75.0 (16.0) |  |
| **Gender (%)** |  |  | <0.001 |
| Male | 8503 (39.9) | 2677 (51.8) |  |
| Female | 12810 (60.1) | 2487 (48.2) |  |
| **Category of residence areas (%)** |  |  | <0.001 |
| City | 4817 (22.6) | 851 (16.5) |  |
| Town | 3949 (18.5) | 957 (18.5) |  |
| Rural | 12547 (58.9) | 3356 (65.0) |  |
| **Living pattern (%)** |  |  | 0.879 |
| Living with family member(s) | 18268 (85.7) | 4429 (85.9) |  |
| Living alone | 3028 (14.2) | 728 (14.1) |  |
| **Education (years)** |  |  | <0.001 |
| median (interquartile range) | 0.0 (3.0) | 1.0 (5.0) |  |
| **Self-reported economic status (%)** |  |  | <0.001 |
| Very poor | 725 (3.4) | 98 (1.9) |  |
| Poor | 3166 (14.9) | 643 (12.5) |  |
| Fair | 14134 (66.3) | 3554 (68.9) |  |
| Rich | 2895 (13.6) | 801 (15.5) |  |
| Very rich | 247 (1.2) | 59 (1.1) |  |
| **Smoking (%)** |  |  | <0.001 |
| Yes | 3526 (16.5) | 1355 (26.2) |  |
| No | 17780 (83.4) | 3807 (73.8) |  |
| **Drinking (%)** |  |  | <0.001 |
| Yes | 3794 (17.8) | 1277 (24.7) |  |
| No | 17513 (82.2) | 3883 (75.3) |  |
| **Regular exercise (%)** |  |  | <0.001 |
| Current | 5407 (25.4) | 1816 (35.2) |  |
| Past | 2775 (13.0) | 371 (7.2) |  |
| Never | 13084 (61.4) | 2964 (57.4) |  |
| **Self-reported sleep quality (%)** |  |  | <0.001 |
| Very bad | 205 (1.0) | 38 (0.7) |  |
| Bad | 1951 (9.2) | 479 (9.3) |  |
| Fair | 5436 (25.5) | 1107 (21.4) |  |
| Good | 10804 (50.7) | 2711 (52.5) |  |
| Very good | 2823 (13.2) | 829 (16.1) |  |
| **Fresh fruit consumption (%)** |  |  | <0.001 |
| rarely or never | 6008 (28.2) | 1033 (20.0) |  |
| occasionally | 7552 (35.4) | 1974 (38.2) |  |
| quite often | 5326 (25.0) | 1534 (29.7) |  |
| almost everyday | 2425 (11.4) | 623 (12.1) |  |
| **Fresh vegetable consumption (%)** |  |  | <0.001 |
| rarely or never | 916 (4.3) | 62 (1.2) |  |
| occasionally | 2341 (11.0) | 385 (7.5) |  |
| quite often | 6996 (32.8) | 1641 (31.8) |  |
| almost everyday | 11054 (51.9) | 3071 (59.5) |  |
| **Hypertension (%)** |  |  | 0.121 |
| Yes | 5646 (26.5) | 1356 (26.6) |  |
| No | 14726 (69.1) | 3736 (73.4) |  |
| **Diabetes (%)** |  |  | <0.001 |
| Yes | 2449 (11.5) | 358 (7.0) |  |
| No | 17812 (83.6) | 4735 (93.0) |  |
| **Heart disease (%)** |  |  | <0.001 |
| Yes | 3521 (16.5) | 640 (12.5) |  |
| No | 16804 (78.8) | 4460 (87.5) |  |
| **Cerebrovascular disease (%)** |  |  | <0.001 |
| Yes | 2721 (12.8) | 355 (7.0) |  |
| No | 17587 (82.5) | 4741 (93.0) |  |
| **Cancer (%)** |  |  | <0.001 |
| Yes | 1456 (6.8) | 89 (1.8) |  |
| No | 18667 (87.6) | 4969 (98.2) |  |

Data are expressed as numbers (percentages) or median (interquartile range).

**Supplementary Table 4 Fit indices for two- to six-class growth mixture models for SBP.**

| Class | LOGLIK | BIC | AIC | 2*ΔBIC | The size of the smallest class | Mean posterior probabilities |
| --- | --- | --- | --- | --- | --- | --- |
| 2 traj-x^2^ | -76444.54 | -76478.73 | -76452.54 | —— | 807 (15.63%) | 0.92/0.80 |
| 3 traj-x^2^ | -76382.16 | -76433.46 | -76394.16 | 90.54 | 78 (1.51%) | 0.72/0.87/0.77 |
| **4 traj-x^2^** | **-76337.38** | **-76405.78** | **-76353.38** | **55.36** | **50 (0.97%)** | **0.66/0.81/0.71/0.76** |
| 5 traj-x^2^ | -76337.38 | -76422.87 | -76357.38 | 34.18 | —— | —— |
| 6 traj-x^2^ | -76325.48 | -76428.07 | -76349.48 | 10.40 | 1 (0.02%) | 0.78/0.56/0.48/0.78/0.63/1.00 |

SBP, systolic blood pressure; LOGLIK, log-likelihood; AIC, Akaike information criterion; BIC, Bayesian information criterion; 2*ΔBIC, the change of the BIC (compared to the preceding BIC) multiplied two.

**Supplementary Table 5 Fit indices for two- to six-class growth mixture models for DBP.**

| Class | LOGLIK | BIC | AIC | 2*ΔBIC | The size of the smallest class | Mean posterior probabilities |
| --- | --- | --- | --- | --- | --- | --- |
| 2 traj-x^2^ | -66317.51 | -66351.71 | -66325.51 | —— | 656 (12.70%) | 0.89/0.75 |
| **3 traj-x^2^** | **-66279.85** | **-66331.14** | **-66291.85** | **41.14** | **297 (5.75%)** | **0.82/0.66/0.74** |
| 4 traj-x^2^ | -66264.63 | -66333.03 | -66280.63 | 3.78 | 119 (2.30%) | 0.73/0.61/0.62/0.69 |
| 5 traj-x^2^ | -66247.57 | -66333.06 | -66267.57 | 0.06 | 32 (0.62%) | 0.69/0.61/0.64/0.62/0.69 |
| 6 traj-x^2^ | -66243.06 | -66345.66 | -66267.06 | 25.20 | 3 (0.06%) | 0.63/0.69/0.68/0.63/0.70/0.60 |

DBP, diastolic blood pressure; LOGLIK, log-likelihood; AIC, Akaike information criterion; BIC, Bayesian information criterion; 2*ΔBIC, the change of the BIC (compared to the preceding BIC) multiplied two.

**Supplementary Table 6 Fit indices for two- to six-class growth mixture models for BMI.**

| Class | LOGLIK | BIC | AIC | 2*ΔBIC | The size of the smallest class | Mean posterior probabilities |
| --- | --- | --- | --- | --- | --- | --- |
| 2 traj-x^2^ | -45813.02 | -45847.22 | -45821.02 | —— | 1175 (22.75%) | 0.94/0.88 |
| 3 traj-x^2^ | -45383.18 | -45434.47 | -45395.18 | 825.50 | 327 (6.33%) | 0.88/0.83/0.86 |
| **4 traj-x^2^** | **-45296.07** | **-45364.46** | **-45364.46** | **140.02** | **71 (1.37%)** | **0.87/0.80/0.78/0.84** |
| 5 traj-x^2^ | -45275.80 | -45361.30 | -45295.80 | 6.32 | 54 (1.05%) | 0.67/0.80/0.75/0.63/0.85 |
| 6 traj-x^2^ | -45161.36 | -45263.95 | -45185.36 | 194.70 | 14 (0.27%) | 0.87/0.86/0.80/0.89/0.83/0.89 |

BMI, body mass index; LOGLIK, log-likelihood; AIC, Akaike information criterion; BIC, Bayesian information criterion; 2*ΔBIC, the change of the BIC (compared to the preceding BIC) multiplied two.

**Supplementary Table 7 Fit indices for two- to six-class growth mixture models for MAP.**

| Class | LOGLIK | BIC | AIC | 2*ΔBIC | The size of the smallest class | Mean posterior probabilities |
| --- | --- | --- | --- | --- | --- | --- |
| 2 traj-x^2^ | -67728.01 | -67762.21 | -67736.01 | —— | 808 (15.64%) | 0.91/0.79 |
| **3 traj-x^2^** | **-67679.66** | **-67730.96** | **-67691.66** | **62.50** | **455 (8.81%)** | **0.68/0.79/0.78** |
| 4 traj-x^2^ | -67663.05 | -67731.45 | -67731.45 | 0.98 | 15 (0.29%) | 0.82/0.75/0.67/0.81 |
| 5 traj-x^2^ | -67631.66 | -67717.16 | -67651.66 | 28.58 | 14 (0.27%) | 0.76/0.63/0.64/0.69/0.76 |
| 6 traj-x^2^ | -67620.14 | -67722.73 | -67644.14 | 11.14 | 9 (0.17%) | 0.60/0.63/0.69/0.55/0.63/0.78 |

MAP, mean arterial pressure; LOGLIK, log-likelihood; AIC, Akaike information criterion; BIC, Bayesian information criterion; 2*ΔBIC, the change of the BIC (compared to the preceding BIC) multiplied two.

**Supplementary Table 8 Fit indices for two- to six-class growth mixture models for PP.**

| Class | LOGLIK | BIC | AIC | 2*ΔBIC | The size of the smallest class, | Mean posterior probabilities |
| --- | --- | --- | --- | --- | --- | --- |
| 2 traj-x^2^ | -73812.05 | -73846.25 | -73820.05 | —— | 462 (8.95%) | 0.79/0.95 |
| **3 traj-x^2^** | **-73758.45** | **-73809.75** | **-73770.45** | **73.00** | **48 (0.93%)** | **0.71/0.93/0.69** |
| 4 traj-x^2^ | -73738.99 | -73807.38 | -73754.99 | 4.74 | 26 (0.50%) | 0.68/0.76/0.86/0.66 |
| 5 traj-x^2^ | -73728.91 | -73814.41 | -73748.91 | 14.06 | 12 (0.23%) | 0.67/0.87/0.67/0.67/0.79 |
| 6 traj-x^2^ | -73724.83 | -73827.43 | -73748.83 | 26.04 | 1 (0.02%) | 0.66/0.88/0.64/0.99/0.67/0.88 |

PP, pulse pressure; LOGLIK, log-likelihood; AIC, Akaike information criterion; BIC, Bayesian information criterion; 2*ΔBIC, the change of the BIC (compared to the preceding BIC) multiplied two.

**Supplementary Table 9 Comparison of four group trajectory model shapes in SBP.**

| SBP | LOGLIK | BIC | AIC | 2*ΔBIC |
| --- | --- | --- | --- | --- |
| 4 traj- (2,2,2,2) | -76337.38 | -76405.78 | -76353.38 | Ref. |
| 4 traj- (2,2,2,3) | -76333.41 | -76406.08 | -76350.41 | 0.60 |
| 4 traj- (2,2,3,2) | -76335.08 | -76407.75 | -76352.08 | 3.94 |
| 4 traj- (2,3,2,2) | -76333.41 | -76406.08 | -76350.41 | 0.60 |
| 4 traj- (3,2,2,2) | -76333.41 | -76406.08 | -76350.41 | 0.60 |
| 4 traj- (2,2,3,3) | —— | —— | —— | —— |
| 4 traj- (3,2,3,3) | -76330.93 | -76412.15 | -76349.93 | 12.74 |
| 4 traj- (3,3,2,3) | -76330.65 | -76411.86 | -76349.65 | 12.16 |
| **4 traj- (2,3,2,3)** | **-76337.09** | **-76414.04** | **-76355.09** | **16.52** |
| 4 traj- (3,2,2,3) | -76330.73 | -76407.68 | -76348.73 | 3.80 |
| 4 traj- (2,3,3,2) | -76330.73 | -76407.68 | -76348.73 | 3.80 |
| 4 traj- (3,2,3,2) | -76330.73 | -76407.68 | -76348.73 | 3.80 |
| 4 traj- (3,3,2,2) | -76334.71 | -76411.66 | -76352.71 | 11.76 |
| 4 traj- (2,3,3,3) | -76330.92 | -76412.14 | -76349.92 | 12.72 |
| 4 traj- (3,3,3,2) | -76330.43 | -76411.65 | -76349.43 | 11.74 |
| 4 traj- (3,3,3,3) | -76326.86 | -76412.35 | -76346.86 | 13.14 |

SBP, systolic blood pressure; LOGLIK, log-likelihood; AIC, Akaike information criterion; BIC, Bayesian information criterion; 2*ΔBIC, the change of the BIC (compared to the preceding BIC) multiplied two.

**Supplementary Table 10 Comparison of three group trajectory model shapes in DBP.**

| DBP | LOGLIK | BIC | AIC | 2*ΔBIC |
| --- | --- | --- | --- | --- |
| 3 traj- (2,2,2) | -66279.85 | -66331.14 | -66291.85 | Ref. |
| 3 traj- (2,2,3) | -66279.83 | -66335.40 | -66292.83 | 8.52 |
| 3 traj- (2,3,2) | -66279.19 | -66334.76 | -66292.19 | 7.24 |
| 3 traj- (3,2,2) | -66279.19 | -66334.76 | -66292.19 | 7.24 |
| 3 traj- (3,3,2) | -66278.35 | -66338.19 | -66292.35 | 14.10 |
| 3 traj- (2,3,3) | -66279.16 | -66339.00 | -66293.16 | 15.72 |
| 3 traj- (3,2,3) | -66278.57 | -66338.41 | 66292.57 | 14.54 |
| **3 traj- (3,3,3)** | **-66278.35** | **-66342.47** | **-66293.35** | **22.66** |

DBP, diastolic blood pressure; LOGLIK, log-likelihood; AIC, Akaike information criterion; BIC, Bayesian information criterion; 2*ΔBIC, the change of the BIC (compared to the preceding BIC) multiplied two.

**Supplementary Table 11 Comparison of four group trajectory model shapes in BMI.**

| BMI | LOGLIK | BIC | AIC | 2*ΔBIC |
| --- | --- | --- | --- | --- |
| 4 traj- (2,2,2,2) | -45296.07 | -45364.46 | -45364.46 | Ref. |
| 4 traj- (2,2,3,2) | -45293.00 | -45365.67 | -45310.00 | 2.42 |
| 4 traj- (2,2,2,3) | -45295.88 | -45368.55 | -45312.88 | 8.18 |
| 4 traj- (2,3,2,2) | -45285.78 | -45358.45 | -45302.78 | 12.02 |
| 4 traj- (3,2,2,2) | -45294.16 | -45366.83 | -45311.16 | 4.74 |
| 4 traj- (2,2,3,3) | -45253.70 | -45330.64 | -45271.70 | 67.64 |
| 4 traj- (3,2,3,3) | -45253.10 | -45334.32 | -45272.10 | 60.28 |
| **4 traj- (3,3,2,3)** | **-45243.84** | **-45325.06** | **-45262.84** | **78.80** |
| 4 traj- (2,3,2,3) | -45293.94 | -45370.88 | -45311.94 | 12.84 |
| 4 traj- (3,2,2,3) | -45253.70 | -45330.64 | -45271.70 | 67.64 |
| 4 traj- (2,3,3,2) | -45291.02 | -45367.96 | -45309.02 | 7.00 |
| 4 traj- (3,2,3,2) | -45282.91 | -45359.86 | -45300.91 | 9.20 |
| 4 traj- (3,3,2,2) | -45282.91 | -45359.86 | -45300.91 | 9.20 |
| 4 traj- (2,3,3,3) | -45253.10 | -45334.32 | -45272.10 | 60.28 |
| 4 traj- (3,3,3,2) | —— | —— | —— | —— |
| 4 traj- (3,3,3,3) | -45243.04 | -45328.54 | -45263.04 | 71.84 |

BMI, body mass index; LOGLIK, log-likelihood; AIC, Akaike information criterion; BIC, Bayesian information criterion; 2*ΔBIC, the change of the BIC (compared to the preceding BIC) multiplied two.

**Supplementary Table 12 Comparison of three group trajectory model shapes in MAP.**

| MAP | LOGLIK | BIC | AIC | 2*ΔBIC |
| --- | --- | --- | --- | --- |
| 3 traj- (2,2,2) | -67679.66 | -67730.96 | -67691.66 | Ref. |
| 3 traj- (2,2,3) | -67678.30 | -67733.87 | -67691.30 | 5.82 |
| 3 traj- (2,3,2) | -67677.14 | -67732.71 | -67690.14 | 3.50 |
| 3 traj- (3,2,2) | -67677.14 | -67732.71 | -67690.14 | 3.50 |
| 3 traj- (3,3,2) | -67677.08 | -67736.93 | -67691.08 | 11.94 |
| 3 traj- (2,3,3) | -67675.46 | -67735.30 | -67689.46 | 8.68 |
| 3 traj- (3,2,3) | -67675.46 | -67735.30 | -67689.46 | 8.68 |
| **3 traj- (3,3,3)** | **-67675.26** | **-67739.38** | **-67690.26** | **16.84** |

MAP, mean arterial pressure; LOGLIK, log-likelihood; AIC, Akaike information criterion; BIC, Bayesian information criterion; 2*ΔBIC, the change of the BIC (compared to the preceding BIC) multiplied two.

**Supplementary Table 13 Comparison of three group trajectory model shapes in PP.**

| PP | LOGLIK | BIC | AIC | 2*ΔBIC |
| --- | --- | --- | --- | --- |
| 3 traj- (2,2,2) | -73758.45 | -73809.75 | -73770.45 | Ref. |
| 3 traj- (2,2,3) | -73758.25 | -73813.82 | -73771.25 | 8.14 |
| 3 traj- (2,3,2) | -73762.20 | -73817.77 | -73775.20 | 16.04 |
| 3 traj- (3,2,2) | -73773.23 | -73828.80 | -73786.23 | 38.10 |
| 3 traj- (3,3,2) | -73773.13 | -73832.98 | -73787.13 | 46.46 |
| 3 traj- (2,3,3) | 73763.22 | -73823.06 | -73777.22 | 26.62 |
| 3 traj- (3,2,3) | -73772.93 | -73832.78 | -73786.93 | 46.06 |
| **3 traj- (3,3,3)** | **-73772.52** | **-73836.64** | **-73787.52** | **53.78** |

PP, pulse pressure; LOGLIK, log-likelihood; AIC, Akaike information criterion; BIC, Bayesian information criterion; 2*ΔBIC, the change of the BIC (compared to the preceding BIC) multiplied two.

**Supplementary Table 14 Posterior probabilities for CVRFs trajectories.**

| Trajectory Model | Class | N (%) | Mean (SD) |
| --- | --- | --- | --- |
| 4 traj- (2,3,2,3) for SBP | High stable SBP | 1046 (20.26) | 0.66 (0.15) |
|  | Normal stable SBP | 3863 (74.81) | 0.81 (0.14) |
|  | Lowered SBP | 204 (3.95) | 0.71 (0.19) |
|  | Greatly elevated SBP | 51 (0.99) | 0.75 (0.20) |
| 3 traj- (3,3,3) for DBP | Normal stable DBP | 4472 (86.60) | 0.81 (0.11) |
|  | Low stable DBP | 394 (7.63) | 0.66 (0.12) |
|  | High stable DBP | 298 (5.77) | 0.75 (0.15) |
| 4 traj- (3,3,2,3) for BMI | Progressively obese | 362 (7.01) | 0.84 (0.14) |
|  | Stable slim | 2872 (55.62) | 0.87 (0.14) |
|  | Stable normal weight | 1881 (36.43) | 0.80 (0.15) |
|  | Lowered weight | 49 (0.95) | 0.91 (0.13) |
| 3 traj- (3,3,3) for MAP | Low stable MAP | 483 (9.35) | 0.68 (0.12) |
|  | Normal stable MAP | 4196 (81.25) | 0.80 (0.11) |
|  | High stable MAP | 485 (9.39) | 0.78 (0.16) |
| 3 traj- (3,3,3) for PP | Elevated PP | 385 (7.46) | 0.73 (0.18) |
|  | Normal stable PP | 4668 (90.40) | 0.90 (0.11) |
|  | Lowered PP | 111 (2.15) | 0.68 (0.19) |

SBP, systolic blood pressure; DBP, diastolic blood pressure; BMI, body mass index; MAP, mean arterial pressure; PP, pulse pressure. Values are means (standard deviations) for posterior probabilities, and numbers (percentages) for categorical variables.

**Supplementary Table 15 Parameter estimation results.**

| Class | Intercept | Linear | Quadratic | Cubic |
| --- | --- | --- | --- | --- |
| High stable SBP | -94.21 (38.35) | 5.44 (0.91) | -0.03 (0.01) | —— |
| Normal stable SBP | 71.55 (99.85) | 1.44 (3.64) | -0.01 (0.04) | 0.00002 (0.00018) |
| Lowered SBP | 337.60 (68.99) | -3.48 (1.66) | 0.01 (0.01) | —— |
| Greatly elevated SBP | 166.35 (989.68) | -9.70 (36.60) | 0.22 (0.45) | -0.00125 (0.00181) |
| Normal stable DBP | 26.89 (54.21) | 2.24 (1.97) | -0.03 (0.02) | 0.00012 (0.00009) |
| Low stable DBP | -20.36 (160.04) | 3.72 (5.88) | -0.05 (0.07) | 0.00018 (0.00029) |
| High stable DBP | 131.88 (183.90) | -0.79 (6.78) | 0.003 (0.083) | 0.00001 (0.00033) |
| Progressively obese | 242.12 (54.52) | -8.58 (2.05) | 0.11 (0.03) | -0.00048 (0.00010) |
| Stable slim | -63.33 (17.10) | 2.99 (0.62) | -0.04 (0.01) | 0.00014 (0.00003) |
| Stable normal weight | 11.54 (3.13) | 0.30 (0.08) | -0.0019 (0.0005) | —— |
| Lowered weight | -1087.78 (111.58) | 43.28 (4.24) | -0.55 (0.05) | 0.00227 (0.00022) |
| Low stable MAP | 157.05 (159.13) | -2.70 (5.85) | 0.04 (0.07) | -0.00016 (0.00029) |
| Normal stable MAP | -66.59 (65.74) | 5.86 (2.42) | -0.07 (0.03) | 0.00026 (0.00012) |
| High stable MAP | 479.53 (206.24) | -13.33 (7.73) | 0.16 (0.10) | -0.00063 (0.00039) |
| Elevated PP | 394.33 (301.93) | -16.62 (11.04) | 0.25 (0.13) | -0.00117 (0.00053) |
| Normal stable PP | 32.30 (88.39) | -0.44 (3.28) | 0.02 (0.04) | -0.00011 (0.00016) |
| Lowered PP | -117.77 (574.46) | 10.39 (21.91) | -0.16 (0.28) | 0.00072 (0.00115) |

SBP, systolic blood pressure; DBP, diastolic blood pressure; BMI, body mass index; MAP, mean arterial pressure; PP, pulse pressure. Values are means (standard error) for parameter estimation.

**Supplementary Table 16 Subgroup analyses of the effects of SBP trajectory on the risk of cognitive impairment.**

| Subgroup | High stable SBP | Greatly elevated SBP | Lowered SBP | P-interaction |
| --- | --- | --- | --- | --- |
| **Gender** |  |  |  | 0.73 |
| Male | 0.98 (0.81, 1.18) | 0.70 (0.29, 1.71) | 2.04 (1.27, 3.27)* |  |
| Female | 0.87 (0.74, 1.02) | 0.79 (0.44, 1.42) | 1.42 (0.94, 2.15) |  |
| **Category of residence areas** |  |  |  | 0.07 |
| City | 0.95 (0.66, 1.37) | 0.00 (0.00, 0.00) | 0.70 (0.27, 1.81) |  |
| Town | 0.87 (0.66, 1.15) | 1.36 (0.48, 3.82) | 3.87 (1.87, 7.99)* |  |
| Rural | 0.91 (0.78, 1.05) | 0.67 (0.38, 1.17) | 1.73 (1.19, 2.51)* |  |
| **Living pattern** |  |  |  | 0.03 |
| Living with family member(s) | 0.91 (0.80, 1.04) | 0.87 (0.51, 1.49) | 1.48 (1.07, 2.05)* |  |
| Living alone | 0.95 (0.72, 1.26) | 0.52 (0.16, 1.71) | 7.73 (2.72, 21.98)* |  |

SBP, systolic blood pressure. Hazard ratios (95% confidence intervals) are presented when normal stable SBP is used as reference. The estimated effects were based on the model 4. The interaction terms tests whether the association between SBP trajectory and cognitive impairment differs as a function of gender, residence areas, and living pattern. * P <0.05

**Supplementary Table 17 Subgroup analyses of the effects of DBP trajectory on the risk of cognitive impairment.**

| Subgroup | High stable DBP | Low stable DBP | P-interaction |
| --- | --- | --- | --- |
| **Gender** |  |  | 0.03 |
| Male | 0.76 (0.53, 1.11) | 0.63 (0.47, 0.85)* |  |
| Female | 1.20 (0.94, 1.52) | 0.91 (0.72, 1.15) |  |
| **Category of residence areas** |  |  | 0.23 |
| City | 0.66 (0.35, 1.23) | 0.47 (0.29, 0.78)* |  |
| Town | 1.03 (0.63, 1.67) | 0.91 (0.59, 1.41) |  |
| Rural | 1.08 (0.85, 1.38) | 0.85 (0.68, 1.06) |  |
| **Living pattern** |  |  | 0.62 |
| Living with family member(s) | 0.97 (0.77, 1.22) | 0.78 (0.64, 0.95)* |  |
| Living alone | 1.23 (0.80, 1.90) | 0.90 (0.53, 1.53) |  |

DBP, diastolic blood pressure. Hazard ratios (95% confidence intervals) are presented when normal stable DBP is used as reference. The estimated effects were based on the model 4. The interaction terms tests whether the association between DBP trajectory and cognitive impairment differs as a function of gender, residence areas, and living pattern. * P <0.05.

**Supplementary Table 18 Subgroup analyses of the effects of BMI trajectory on the risk of cognitive impairment.**

| Subgroup | Progressively obese | Stable slim | Lowered weight | P-interaction |
| --- | --- | --- | --- | --- |
| **Gender** |  |  |  | 0.63 |
| Male | 1.31 (0.89, 1.93) | 1.19 (1.02, 1.39)* | 2.30 (0.72, 7.30) |  |
| Female | 1.31 (0.98, 1.75) | 1.10 (0.96, 1.26) | 1.08 (0.55, 2.12) |  |
| **Category of residence areas** |  |  |  | 0.08 |
| City | 0.70 (0.39, 1.28) | 1.06 (0.82, 1.38) | 4.00 (0.94, 17.09) |  |
| Town | 2.05 (1.24, 3.37)* | 1.41 (1.11, 1.80)* | 1.72 (0.60, 4.91) |  |
| Rural | 1.37 (1.02, 1.84)* | 1.10 (0.96, 1.24) | 0.87 (0.38, 1.99) |  |
| **Living pattern** |  |  |  | 0.43 |
| Living with family member(s) | 1.27 (0.99, 1.63) | 1.14 (1.02, 1.28)* | 1.69 (0.87, 3.29) |  |
| Living alone | 1.20 (0.64, 2.26) | 1.16 (0.90, 1.50) | 0.49 (0.14, 1.74) |  |

BMI, body mass index. Hazard ratios (95% confidence intervals) are presented when stable normal weight is used as reference. The estimated effects were based on the model 4. The interaction terms tests whether the association between BMI trajectory and cognitive impairment differs as a function of gender, residence areas, and living pattern. * P <0.05

**Supplementary Table 19 Subgroup analyses of the effects of MAP trajectory on the risk of cognitive impairment.**

| Subgroup | Low stable MAP | High stable MAP | P-interaction |
| --- | --- | --- | --- |
| **Gender** |  |  | 0.50 |
| Male | 0.92 (0.72, 1.18) | 0.92 (0.70, 1.21) |  |
| Female | 1.07 (0.87, 1.32) | 1.08 (0.89, 1.33) |  |
| **Category of residence areas** |  |  | 0.19 |
| City | 0.72 (0.48, 1.07) | 0.74 (0.42, 1.30) |  |
| Town | 1.12 (0.76, 1.65) | 1.17 (0.82, 1.67) |  |
| Rural | 1.06 (0.87, 1.29) | 1.01 (0.83, 1.23) | 0.14 |
| **Living pattern** |  |  |  |
| Living with family member(s) | 0.95 (0.80, 1.13) | 1.04 (0.87, 1.24) |  |
| Living alone | 1.45 (0.97, 2.16) | 0.99 (0.67, 1.45) |  |

MAP, mean arterial pressure. Hazard ratios (95% confidence intervals) are presented when normal stable MAP is used as reference. The estimated effects were based on the model 4. The interaction terms tests whether the association between MAP trajectory and cognitive impairment differs as a function of gender, residence areas, and living pattern. * P <0.05

**Supplementary Table 20 Subgroup analyses of the effects of PP trajectory on the risk of cognitive impairment.**

| Subgroup | Elevated PP | Lowered PP | P-interaction |
| --- | --- | --- | --- |
| **Gender** |  |  | 0.35 |
| Male | 0.68 (0.49, 0.94)* | 3.63 (1.96, 6.71)* |  |
| Female | 0.79 (0.62, 1.00) | 1.85 (0.91, 3.76) |  |
| **Category of residence areas** |  |  | 0.38 |
| City | 0.94 (0.54, 1.64) | 2.33 (0.56, 9.72) |  |
| Town | 0.53 (0.33, 0.86)* | 6.13 (2.54, 14.79)* |  |
| Rural | 0.78 (0.62, 0.98)* | 2.26 (1.23, 4.15)* |  |
| **Living pattern** |  |  | 0.50 |
| Living with family member(s) | 0.76 (0.62, 0.94)* | 2.44 (1.50, 3.99)* |  |
| Living alone | 0.76 (0.48, 1.20) | 8.01 (1.77, 36.30)* |  |

PP, pulse pressure. Hazard ratios (95% confidence intervals) are presented when normal stable PP is used as reference. The estimated effects were based on the model 4. The interaction terms tests whether the association between PP trajectory and cognitive impairment differs as a function of gender, residence areas, and living pattern. * P <0.05

**Supplementary Table 21 Sensitivity analyses on the effects of CVRFs trajectories on the risk of moderate/severe cognitive impairment.**

| Variables | Model 1 | Model 2 | Model 3 | Model 4 |
| --- | --- | --- | --- | --- |
| **Normal stable SBP as reference** | |  |  |  |
| High stable SBP | 0.89 (0.76, 1.05) | 0.88 (0.75, 1.04) | 0.89 (0.76, 1.05) | 0.87 (0.73, 1.04) |
| Greatly elevated SBP | 0.56 (0.25, 1.24) | 0.53 (0.24, 1.18) | 0.53 (0.23, 1.18) | 0.54 (0.24, 1.22) |
| Lowered SBP | 1.82 (1.20, 2.76)* | 1.87 (1.23, 2.85)* | 1.92 (1.26, 2.94)* | 1.82 (1.18, 2.80)* |
| **Normal stable DBP as reference** | |  |  |  |
| High stable DBP | 0.92 (0.69, 1.22) | 0.91 (0.68, 1.20) | 0.92 (0.69, 1.22) | 0.96 (0.72, 1.29) |
| Low stable DBP | 0.80 (0.63, 1.02) | 0.82 (0.65, 1.05) | 0.80 (0.62, 1.02) | 0.771 (0.596, 0.997)* |
| **Stable normal weight as reference** | |  |  |  |
| Progressively obese | 0.90 (0.62, 1.29) | 0.89 (0.62, 1.29) | 0.93 (0.64, 1.34) | 0.93 (0.64, 1.35) |
| Stable slim | 1.22 (1.07, 1.39)* | 1.13 (0.99, 1.30) | 1.11 (0.97, 1.28) | 1.12 (0.98, 1.29) |
| Lowered weight | 0.43 (0.11, 1.72) | 0.40 (0.10, 1.61) | 0.41 (0.10, 1.66) | 0.36 (0.09, 1.46) |
| **Normal stable MAP as reference** | |  |  |  |
| Low stable MAP | 1.03 (0.84, 1.27) | 1.03 (0.84, 1.28) | 1.01 (0.82, 1.25) | 0.99 (0.79, 1.23) |
| High stable MAP | 1.07 (0.86, 1.33) | 1.03 (0.83, 1.28) | 1.04 (0.84, 1.29) | 1.05 (0.84, 1.32) |
| **Normal stable PP as reference** | |  |  |  |
| Elevated PP | 0.78 (0.60, 1.01) | 0.77 (0.60, 1.00) | 0.78 (0.60, 1.01) | 0.758 (0.576, 0.997)* |
| Lowered PP | 4.63 (2.52, 8.51)* | 5.01 (2.72, 9.22)* | 4.94 (2.68, 9.13)* | 5.12 (2.76, 9.50)* |

SBP, systolic blood pressure; DBP, diastolic blood pressure; MAP, mean arterial pressure; PP, pulse pressure. Hazard ratios (95% confidence intervals) are presented. Model 1 was adjusted for no covariates. Model 2 was adjusted for gender, category of residence areas, living pattern, education level, and self-reported economic status based on model 1. Model 3 was adjusted for model 2 plus smoking, drinking, regular exercise, self-reported sleep quality, fresh fruit consumption, and vegetable consumption. Model 4 was adjusted for model 3 plus hypertension, diabetes, heart disease, cerebrovascular disease, and cancer. * P <0.05.

**Supplementary Table 22 Sensitivity analyses on the effects of CVRFs trajectories on the risk of severe cognitive impairment.**

| Variables | Model 1 | Model 2 | Model 3 | Model 4 |
| --- | --- | --- | --- | --- |
| **Normal stable SBP as reference** | |  |  |  |
| High stable SBP | 1.01 (0.81, 1.25) | 1.01 (0.82, 1.26) | 1.01 (0.81, 1.26) | 1.01 (0.80, 1.27) |
| Greatly elevated SBP | 0.56 (0.18, 1.75) | 0.58 (0.19, 1.80) | 0.57 (0.18, 1.79) | 0.66 (0.21, 2.09) |
| Lowered SBP | 1.90 (1.07, 3.39)* | 1.87 (1.04, 3.34)* | 1.97 (1.09, 3.56)* | 1.91 (1.05, 3.49)* |
| **Normal stable DBP as reference** | |  |  |  |
| High stable DBP | 0.98 (0.67, 1.42) | 0.97 (0.66, 1.41) | 0.98 (0.67, 1.43) | 1.03 (0.70, 1.52) |
| Low stable DBP | 0.71 (0.50, 1.01) | 0.71 (0.50, 1.00) | 0.68 (0.48, 0.98)* | 0.62 (0.43, 0.90)* |
| **Stable normal weight as reference** | |  |  |  |
| Progressively obese | 0.69 (0.39, 1.21) | 0.70 (0.40, 1.23) | 0.74 (0.42, 1.31) | 0.75 (0.42, 1.32) |
| Stable slim | 1.21 (1.00, 1.45)* | 1.17 (0.98, 1.41) | 1.16 (0.96, 1.39) | 1.18 (0.97, 1.42) |
| Lowered weight | 0.83 (0.21, 3.37) | 0.88 (0.22, 3.56) | 0.90 (0.22, 3.67) | 0.79 (0.19, 3.26) |
| **Normal stable MAP as reference** | |  |  |  |
| Low stable MAP | 1.15 (0.88, 1.52) | 1.12 (0.85, 1.48) | 1.07 (0.81, 1.41) | 0.98 (0.73, 1.31) |
| High stable MAP | 1.18 (0.89, 1.58) | 1.17 (0.88, 1.57) | 1.16 (0.87, 1.56) | 1.20 (0.88, 1.62) |
| **Normal stable PP as reference** | |  |  |  |
| Elevated PP | 0.71 (0.49, 1.04) | 0.71 (0.49, 1.04) | 0.72 (0.49, 1.05) | 0.77 (0.52, 1.13) |
| Lowered PP | 4.84 (1.95, 11.97)* | 5.05 (2.04, 12.51)* | 4.76 (1.91, 11.86)* | 5.30 (2.12, 13.27)* |

SBP, systolic blood pressure; DBP, diastolic blood pressure; MAP, mean arterial pressure; PP, pulse pressure. Hazard ratios (95% confidence intervals) are presented. Model 1 was adjusted for no covariates. Model 2 was adjusted for gender, category of residence areas, living pattern, education level, and self-reported economic status based on model 1. Model 3 was adjusted for model 2 plus smoking, drinking, regular exercise, self-reported sleep quality, fresh fruit consumption, and vegetable consumption. Model 4 was adjusted for model 3 plus hypertension, diabetes, heart disease, cerebrovascular disease, and cancer. * P <0.05.

**Supplementary Table 23 Additional sensitivity analysis of the effect of CVRFs trajectories on the risk of cognitive impairment.**

| Variables | Model 1 | Model 2 | Model 3 | Model 4 |  |
| --- | --- | --- | --- | --- | --- |
| **Excluding 1835 participants with a history of hypertension, diabetes, heart disease, cerebrovascular disease or cancer at baseline** | | | | |  |
| **Normal stable SBP as reference** | |  |  |  |  |
| High stable SBP | 0.95 (0.82, 1.11) | 0.93 (0.79, 1.08) | 0.94 (0.80, 1.10) | 0.94 (0.80, 1.10) |  |
| Greatly elevated SBP | 0.90 (0.34, 2.41) | 0.89 (0.33, 2.39) | 0.83 (0.31, 2.23) | 0.83 (0.31, 2.23) |  |
| Lowered SBP | 1.41 (0.87, 2.29) | 1.35 (0.83, 2.20) | 1.30 (0.80, 2.13) | 1.30 (0.80, 2.13) |  |
| **Normal stable DBP as reference** | |  |  |  |  |
| High stable DBP | 0.85 (0.63, 1.14) | 0.88 (0.65, 1.19) | 0.88 (0.65, 1.20) | 0.88 (0.65, 1.20) |  |
| Low stable DBP | 0.86 (0.70, 1.07) | 0.87 (0.71, 1.08) | 0.83 (0.67, 1.03) | 0.83 (0.67, 1.03) |  |
| **Stable normal weight as reference** | |  |  |  |  |
| Progressively obese | 1.48 (1.09, 1.99)* | 1.48 (1.09, 2.00)* | 1.53 (1.13, 2.08)* | 1.53 (1.13, 2.08)* |  |
| Stable slim | 1.35 (1.19, 1.53)* | 1.26 (1.11, 1.43)* | 1.25 (1.10, 1.42)* | 1.25 (1.10, 1.42)* |  |
| Lowered weight | 2.12 (0.95, 4.76) | 2.14 (0.95, 4.83) | 2.26 (1.00, 5.15) | 2.26 (1.00, 5.15) |  |
| **Normal stable MAP as reference** | |  |  |  |  |
| Low stable MAP | 1.09 (0.92, 1.30) | 1.10 (0.92, 1.31) | 1.08 (0.90, 1.29) | 1.08 (0.90, 1.29) |  |
| High stable MAP | 0.98 (0.77, 1.23) | 0.97 (0.77, 1.22) | 0.97 (0.77, 1.23) | 0.97 (0.77, 1.23) |  |
| **Normal stable PP as reference** | |  |  |  |  |
| Elevated PP | 0.76 (0.58, 0.99) | 0.75 (0.57, 0.98)* | 0.76 (0.58, 0.99)* | 0.76 (0.58, 0.99)* |  |
| Lowered PP | 3.85 (1.90, 7.77)* | 4.08 (2.01, 8.26)* | 3.91 (1.92, 7.93)* | 3.91 (1.92, 7.93)* |  |
| **Imputing missing covariates using multiple imputation** | | | | | |
| **Normal stable SBP as reference** | |  |  |  |  |
| High stable SBP | 0.94 (0.84, 1.06) | 0.93 (0.83, 1.04) | 0.94 (0.84, 1.05) | 0.92 (0.82, 1.04) |  |
| Greatly elevated SBP | 0.82 (0.51, 1.32) | 0.74 (0.46, 1.19) | 0.73 (0.45, 1.19) | 0.92 (0.82, 1.04) |  |
| Lowered SBP | 1.67 (1.24, 2.25)* | 1.71 (1.27, 2.31)* | 1.71 (1.27, 2.30)* | 1.64 (1.21, 2.21)* |  |
| **Normal stable DBP as reference** | |  |  |  |  |
| High stable DBP | 0.98 (0.80, 1.19) | 0.98 (0.81, 1.20) | 0.99 (0.82, 1.21) | 0.99 (0.81, 1.21) |  |
| Low stable DBP | 0.80 (0.67, 0.95)* | 0.83 (0.70, 0.99)* | 0.81 (0.68, 0.97)* | 0.81 (0.68, 0.96)* |  |
| **Stable normal weight as reference** | |  |  |  |  |
| Progressively obese | 1.255 (1.003, 1.570)* | 1.27 (1.01, 1.59)* | 1.30 (1.04, 1.63)* | 1.28 (1.02, 1.60)* |  |
| Stable slim | 1.25 (1.14, 1.38)* | 1.14 (1.04, 1.26)* | 1.13 (1.02, 1.24)* | 1.13 (1.02, 1.24)* |  |
| Lowered weight | 1.46 (0.82, 2.58) | 1.30 (0.73, 2.31) | 1.30 (0.73, 2.32) | 1.17 (0.65, 2.09) |  |
| **Normal stable MAP as reference** | |  |  |  |  |
| Low stable MAP | 1.01 (0.87, 1.17) | 1.03 (0.88, 1.20) | 1.02 (0.87, 1.19) | 1.02 (0.87, 1.19) |  |
| High stable MAP | 1.07 (0.91, 1.24) | 1.03 (0.88, 1.20) | 1.03 (0.88, 1.21) | 1.02 (0.87, 1.19) |  |
| **Normal stable PP as reference** | |  |  |  |  |
| Elevated PP | 0.78 (0.65, 0.93)* | 0.76 (0.64, 0.92)* | 0.77 (0.64, 0.93)* | 0.76 (0.63, 0.92)* |  |
| Lowered PP | 2.58 (1.65, 4.04)* | 2.79 (1.78, 4.37)* | 2.76 (1.76, 4.32)* | 2.68 (1.70, 4.20)* |  |

SBP, systolic blood pressure; DBP, diastolic blood pressure; MAP, mean arterial pressure; PP, pulse pressure. Hazard ratios (95% confidence intervals) are presented. Model 1 was adjusted for no covariates. Model 2 was adjusted for gender, category of residence areas, living pattern, education level, and self-reported economic status based on model 1. Model 3 was adjusted for model 2 plus smoking, drinking, regular exercise, self-reported sleep quality, fresh fruit consumption, and vegetable consumption. Model 4 was adjusted for model 3 plus hypertension, diabetes, heart disease, cerebrovascular disease, and cancer. * P <0.05.


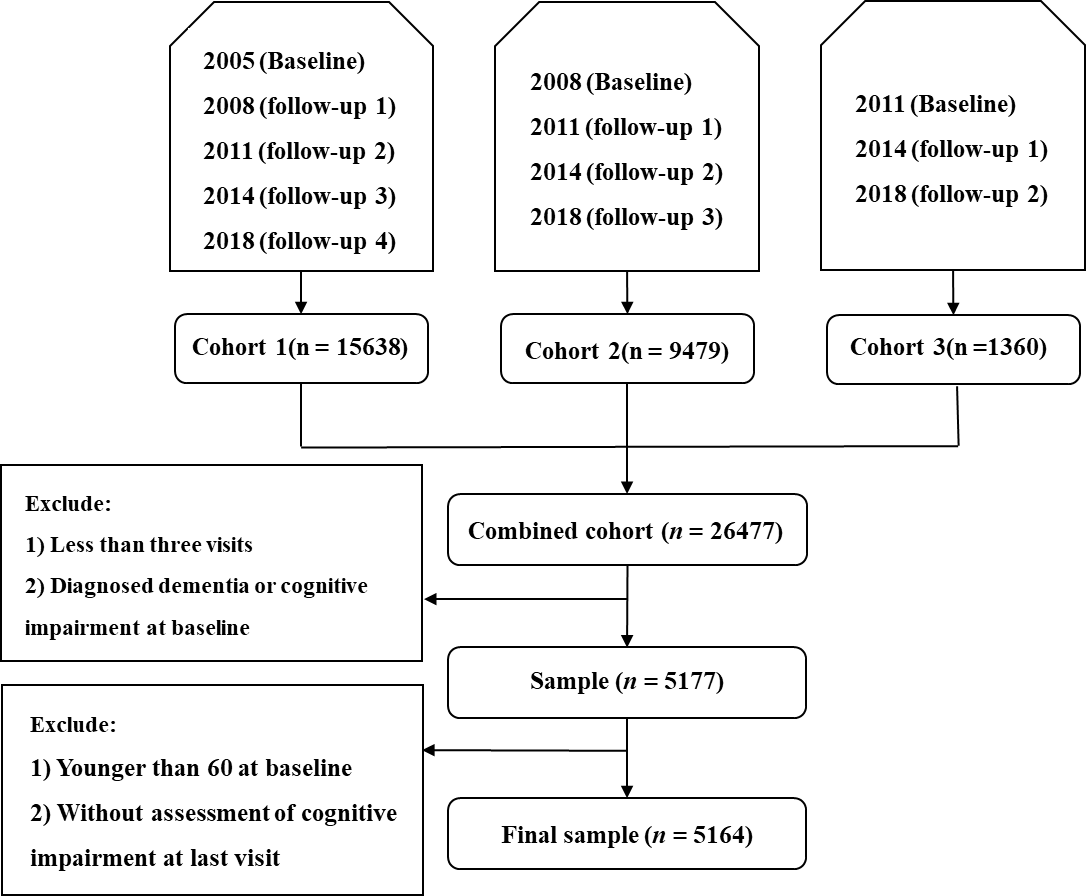


**Supplementary Figure 1.** Recruitment Process

**
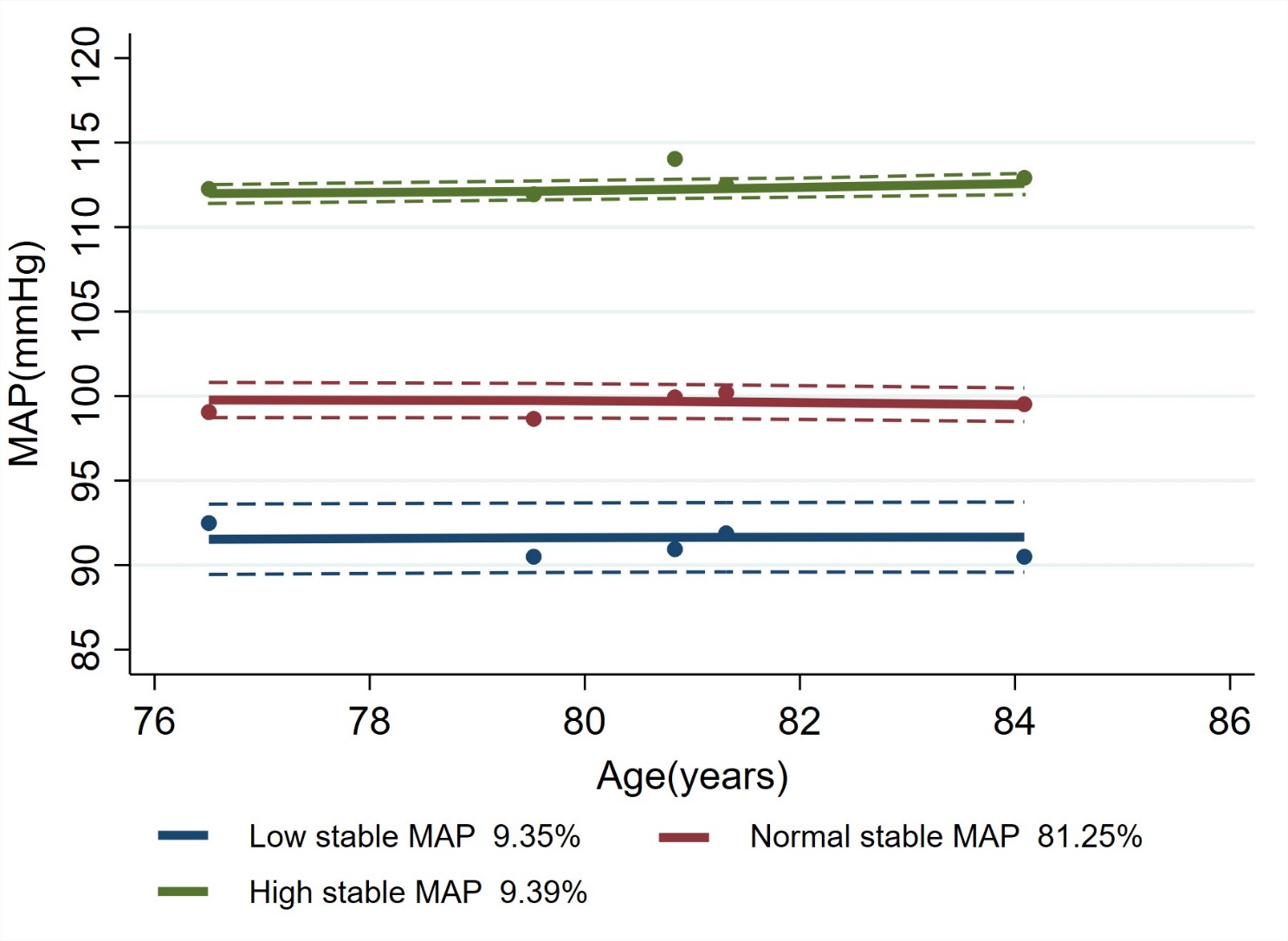
**

**Supplementary Figure 2.** Latent trajectories of MAP for Chinese older people. Notes: Estimated trajectories (solid lines), observed group means for each survey (dot symbols). Dashed lines are approximated 95% pointwise CIs on the estimated trajectories. MAP, mean arterial pressure.

**
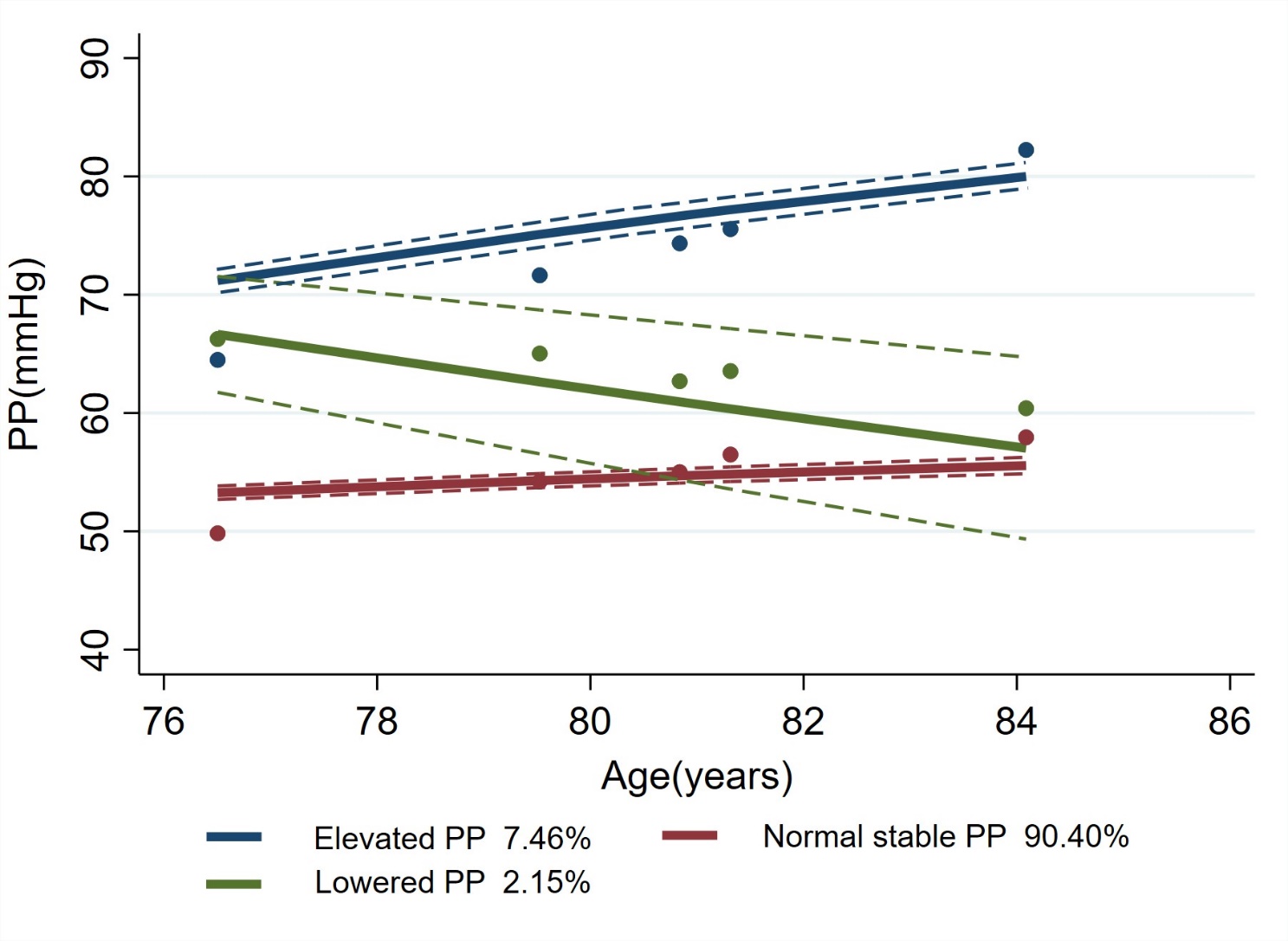
**

**Supplementary Figure 3.** Latent trajectories of PP for Chinese older people. Notes: Estimated trajectories (solid lines), observed group means for each survey (dot symbols). Dashed lines are approximated 95% pointwise CIs on the estimated trajectories. PP, pulse pressure.


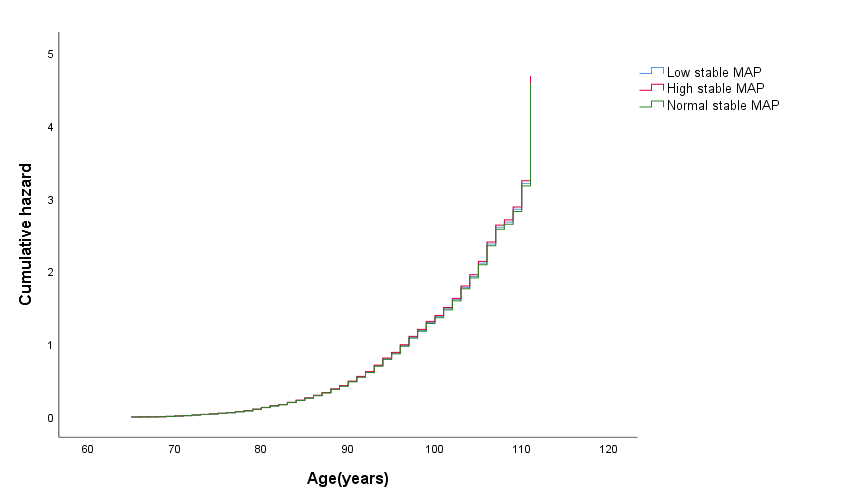


**Supplementary Figure 4.** Curves of the cumulative hazard function of cognitive impairment by trajectory classes of MAP in the final adjusted model. MAP, mean arterial pressure.


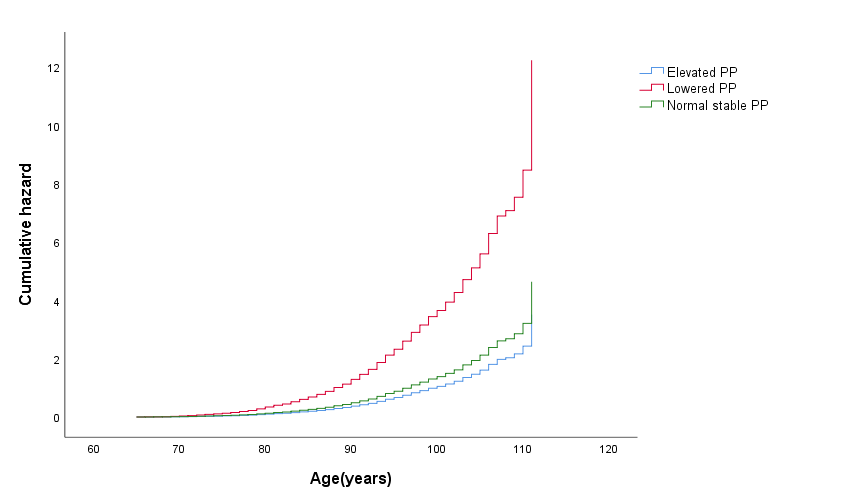


**Supplementary Figure 5.** Curves of the cumulative hazard function of cognitive impairment by trajectory classes of PP in the final adjusted model. PP, pulse pressure.

**
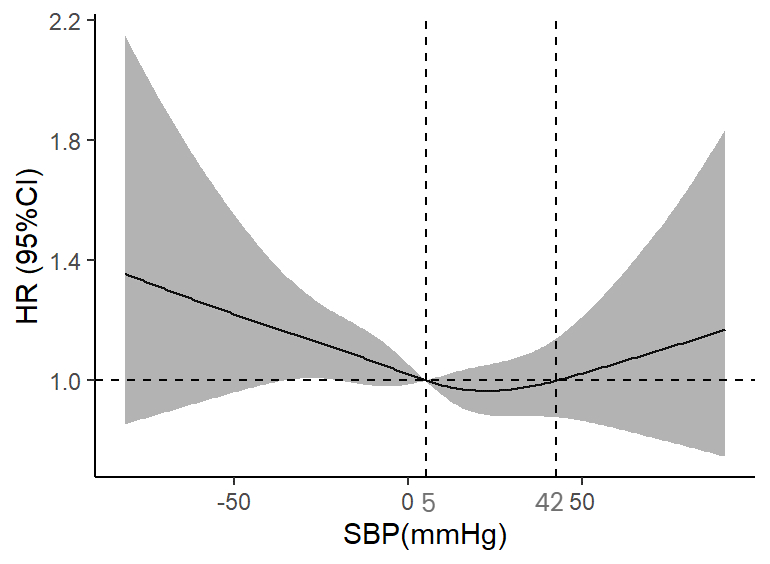
**

**Supplementary Figure 6.** Restricted cubic spline plots of the association of changes in SBP and risk of cognitive impairment. Note. The lines depict the estimated function of SBP for risk of cognitive impairment among the elderly. The shaded gray area indicates the 95% confident interval. SBP, systolic blood pressure;


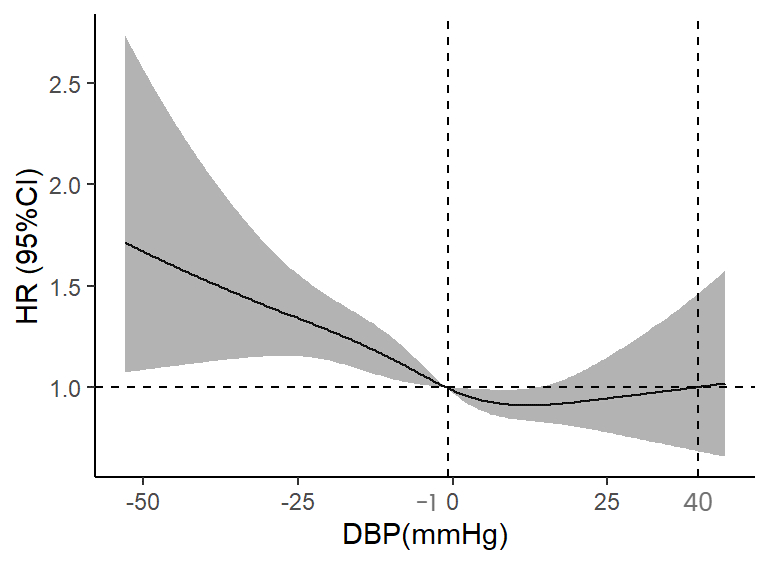


**Supplementary Figure 7.** Restricted cubic spline plots of the association of changes in DBP and risk of cognitive impairment. Note. The lines depict the estimated function of DBP for risk of cognitive impairment among the elderly. The shaded gray area indicates the 95% confident interval. DBP, diastolic blood pressure.


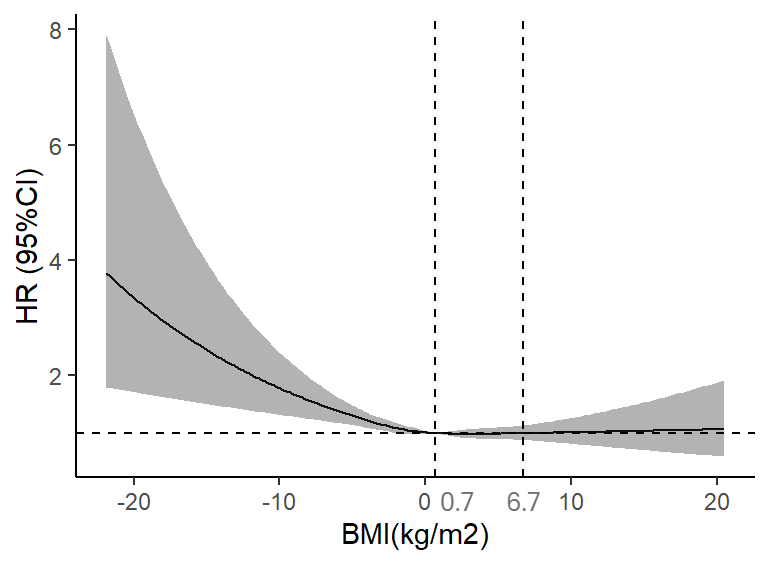


**Supplementary Figure 8.** Restricted cubic spline plots of the association of changes in BMI and risk of cognitive impairment. Note. The lines depict the estimated function of BMI for risk of cognitive impairment among the elderly. The shaded gray area indicates the 95% confident interval. BMI, body mass index.


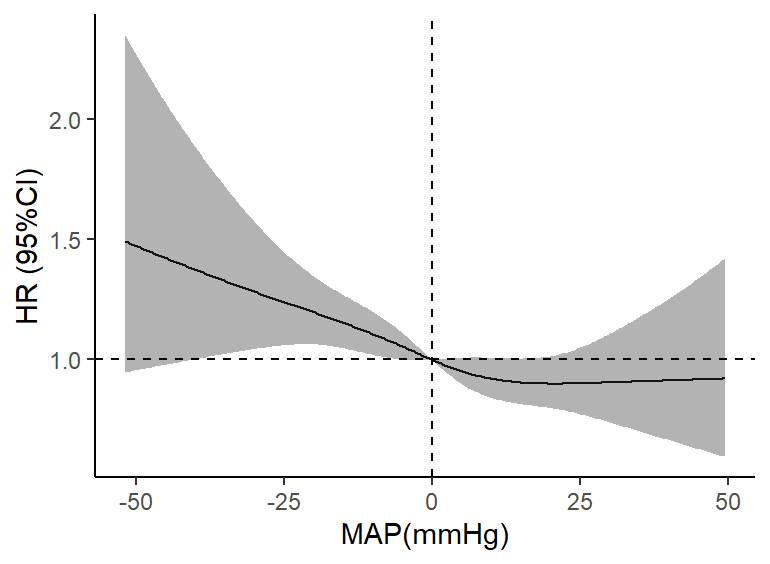


**Supplementary Figure 9.** Restricted cubic spline plots of the association of changes in MAP and risk of cognitive impairment. Note. The lines depict the estimated function of MAP for risk of cognitive impairment among the elderly. The shaded gray area indicates the 95% confident interval. MAP, mean arterial pressure.


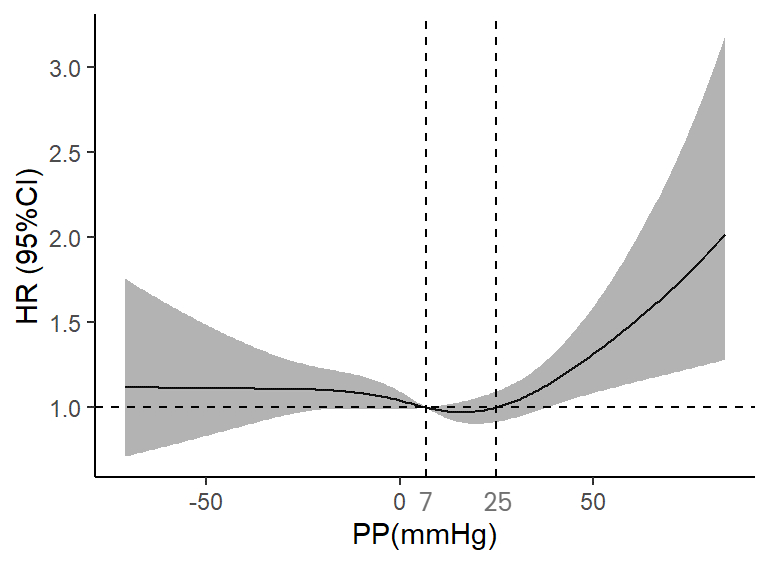


**Supplementary Figure 10.** Restricted cubic spline plots of the association of changes in PP and risk of cognitive impairment. Note. The lines depict the estimated function of PP for risk of cognitive impairment among the elderly. The shaded gray area indicates the 95% confident interval. PP, pulse pressure.

**statistical code**

use "C:\Users\Dang\Documents\mystatafile\trajectory\adultbmi.dta", clear

////GBTM model

describe

traj, var(SBP_*) indep(trueage_*) model(cnorm) min(60) max(250) order(2 2)

tab _traj_Group

traj, var(SBP_*) indep(trueage_*) model(cnorm) min(60) max(250) order(2 2 2)

tab _traj_Group

traj, var(SBP_*) indep(trueage_*) model(cnorm) min(60) max(250) order(2 2 2 2)

tab _traj_Group

traj, var(SBP_*) indep(trueage_*) model(cnorm) min(60) max(250) order(2 2 2 2 2)

tab _traj_Group

traj, var(SBP_*) indep(trueage_*) model(cnorm) min(60) max(250) order(2 2 2 2 2 2)

tab _traj_Group

traj, var(DBP_*) indep(trueage_*) model(cnorm) min(10) max(150) order(2 2)

tab _traj_Group

traj, var(DBP_*) indep(trueage_*) model(cnorm) min(10) max(150) order(2 2 2)

tab _traj_Group

traj, var(DBP_*) indep(trueage_*) model(cnorm) min(10) max(150) order(2 2 2 2)

tab _traj_Group

traj, var(DBP_*) indep(trueage_*) model(cnorm) min(10) max(150) order(2 2 2 2 2)

tab _traj_Group

traj, var(DBP_*) indep(trueage_*) model(cnorm) min(10) max(150) order(2 2 2 2 2 2)

tab _traj_Group

traj, var(BMI_*) indep(trueage_*) model(cnorm) min(8) max(52) order(2 2)

tab _traj_Group

traj, var(BMI_*) indep(trueage_*) model(cnorm) min(8) max(52) order(2 2 2)

tab _traj_Group

traj, var(BMI_*) indep(trueage_*) model(cnorm) min(8) max(52) order(2 2 2 2)

tab _traj_Group

traj, var(BMI_*) indep(trueage_*) model(cnorm) min(8) max(52) order(2 2 2 2 2)

tab _traj_Group

traj, var(BMI_*) indep(trueage_*) model(cnorm) min(8) max(52) order(2 2 2 2 2 2)

tab _traj_Group

traj, var(MAP_*) indep(trueage_*) model(cnorm) min(40) max(170) order(2 2)

tab _traj_Group

traj, var(MAP_*) indep(trueage_*) model(cnorm) min(40) max(170) order(2 2 2)

tab _traj_Group

traj, var(MAP_*) indep(trueage_*) model(cnorm) min(40) max(170) order(2 2 2 2)

tab _traj_Group

traj, var(MAP_*) indep(trueage_*) model(cnorm) min(40) max(170) order(2 2 2 2 2)

tab _traj_Group

traj, var(MAP_*) indep(trueage_*) model(cnorm) min(40) max(170) order(2 2 2 2 2 2)

tab _traj_Group

traj, var(PP_*) indep(trueage_*) model(cnorm) min(0) max(170) order(2 2)

tab _traj_Group

traj, var(PP_*) indep(trueage_*) model(cnorm) min(0) max(170) order(2 2 2)

tab _traj_Group

traj, var(PP_*) indep(trueage_*) model(cnorm) min(0) max(170) order(2 2 2 2)

tab _traj_Group

traj, var(PP_*) indep(trueage_*) model(cnorm) min(0) max(170) order(2 2 2 2 2)

tab _traj_Group

traj, var(PP_*) indep(trueage_*) model(cnorm) min(0) max(170) order(2 2 2 2 2 2)

tab _traj_Group

traj, var(SBP_*) indep(trueage_*) model(cnorm) min(60) max(250) order(2 2 2 2)

traj, var(SBP_*) indep(trueage_*) model(cnorm) min(60) max(250) order(2 2 2 3)

traj, var(SBP_*) indep(trueage_*) model(cnorm) min(60) max(250) order(2 2 3 2)

traj, var(SBP_*) indep(trueage_*) model(cnorm) min(60) max(250) order(2 3 2 2)

traj, var(SBP_*) indep(trueage_*) model(cnorm) min(60) max(250) order(3 2 2 2)

traj, var(SBP_*) indep(trueage_*) model(cnorm) min(60) max(250) order(2 2 3 3)

traj, var(SBP_*) indep(trueage_*) model(cnorm) min(60) max(250) order(2 3 2 3)

traj, var(SBP_*) indep(trueage_*) model(cnorm) min(60) max(250) order(3 2 2 3)

traj, var(SBP_*) indep(trueage_*) model(cnorm) min(60) max(250) order(2 3 3 2)

traj, var(SBP_*) indep(trueage_*) model(cnorm) min(60) max(250) order(3 2 3 2)

traj, var(SBP_*) indep(trueage_*) model(cnorm) min(60) max(250) order(3 3 2 2)

traj, var(SBP_*) indep(trueage_*) model(cnorm) min(60) max(250) order(2 3 3 3)

traj, var(SBP_*) indep(trueage_*) model(cnorm) min(60) max(250) order(3 3 3 2)

traj, var(SBP_*) indep(trueage_*) model(cnorm) min(60) max(250) order(3 3 3 3)

traj, var(SBP_*) indep(trueage_*) model(cnorm) min(60) max(250) order(3 2 3 3)

traj, var(SBP_*) indep(trueage_*) model(cnorm) min(60) max(250) order(3 3 2 3)

traj, var(DBP_*) indep(trueage_*) model(cnorm) min(10) max(150) order(2 2 2)

traj, var(DBP_*) indep(trueage_*) model(cnorm) min(10) max(150) order(2 2 3)

traj, var(DBP_*) indep(trueage_*) model(cnorm) min(10) max(150) order(2 3 2)

traj, var(DBP_*) indep(trueage_*) model(cnorm) min(10) max(150) order(3 2 2)

traj, var(DBP_*) indep(trueage_*) model(cnorm) min(10) max(150) order(3 3 2)

traj, var(DBP_*) indep(trueage_*) model(cnorm) min(10) max(150) order(2 3 3)

traj, var(DBP_*) indep(trueage_*) model(cnorm) min(10) max(150) order(3 2 3)

traj, var(DBP_*) indep(trueage_*) model(cnorm) min(10) max(150) order(3 3 3)

traj, var(BMI_*) indep(trueage_*) model(cnorm) min(8) max(52) order(2 2 2 2)

traj, var(BMI_*) indep(trueage_*) model(cnorm) min(8) max(52) order(2 2 2 3)

traj, var(BMI_*) indep(trueage_*) model(cnorm) min(8) max(52) order(2 2 3 2)

traj, var(BMI_*) indep(trueage_*) model(cnorm) min(8) max(52) order(2 3 2 2)

traj, var(BMI_*) indep(trueage_*) model(cnorm) min(8) max(52) order(3 2 2 2)

traj, var(BMI_*) indep(trueage_*) model(cnorm) min(8) max(52) order(2 2 3 3)

traj, var(BMI_*) indep(trueage_*) model(cnorm) min(8) max(52) order(2 3 2 3)

traj, var(BMI_*) indep(trueage_*) model(cnorm) min(8) max(52) order(3 2 2 3)

traj, var(BMI_*) indep(trueage_*) model(cnorm) min(8) max(52) order(2 3 3 2)

traj, var(BMI_*) indep(trueage_*) model(cnorm) min(8) max(52) order(3 2 3 2)

traj, var(BMI_*) indep(trueage_*) model(cnorm) min(8) max(52) order(3 3 2 2)

traj, var(BMI_*) indep(trueage_*) model(cnorm) min(8) max(52) order(2 3 3 3)

traj, var(BMI_*) indep(trueage_*) model(cnorm) min(8) max(52) order(3 3 3 2)

traj, var(BMI_*) indep(trueage_*) model(cnorm) min(8) max(52) order(3 3 3 3)

traj, var(BMI_*) indep(trueage_*) model(cnorm) min(8) max(52) order(3 2 3 3)

traj, var(BMI_*) indep(trueage_*) model(cnorm) min(8) max(52) order(3 3 2 3)

traj, var(MAP_*) indep(trueage_*) model(cnorm) min(40) max(170) order(2 2 2)

traj, var(MAP_*) indep(trueage_*) model(cnorm) min(40) max(170) order(2 2 3)

traj, var(MAP_*) indep(trueage_*) model(cnorm) min(40) max(170) order(2 3 2)

traj, var(MAP_*) indep(trueage_*) model(cnorm) min(40) max(170) order(3 2 2)

traj, var(MAP_*) indep(trueage_*) model(cnorm) min(40) max(170) order(3 3 2)

traj, var(MAP_*) indep(trueage_*) model(cnorm) min(40) max(170) order(2 3 3)

traj, var(MAP_*) indep(trueage_*) model(cnorm) min(40) max(170) order(3 2 3)

traj, var(MAP_*) indep(trueage_*) model(cnorm) min(40) max(170) order(3 3 3)

traj, var(PP_*) indep(trueage_*) model(cnorm) min(0) max(170) order(2 2 2)

traj, var(PP_*) indep(trueage_*) model(cnorm) min(0) max(170) order(2 2 3)

traj, var(PP_*) indep(trueage_*) model(cnorm) min(0) max(170) order(2 3 2)

traj, var(PP_*) indep(trueage_*) model(cnorm) min(0) max(170) order(3 2 2)

traj, var(PP_*) indep(trueage_*) model(cnorm) min(0) max(170) order(3 3 2)

traj, var(PP_*) indep(trueage_*) model(cnorm) min(0) max(170) order(2 3 3)

traj, var(PP_*) indep(trueage_*) model(cnorm) min(0) max(170) order(3 2 3)

traj, var(PP_*) indep(trueage_*) model(cnorm) min(0) max(170) order(3 3 3)

traj, var(SBP_*) indep(trueage_*) model(cnorm) min(60) max(250) order(2 3 2 3)

trajplot, xtitle(Age) ytitle(SBP) ci xlabel(76(2)86) ylabel(120(10)190)

tab _traj_Group

traj, var(DBP_*) indep(trueage_*) model(cnorm) min(10) max(150) order(3 3 3)

trajplot, xtitle(Age) ytitle(DBP) ci xlabel(76(2)86) ylabel(70(5)100)

tab _traj_Group

traj, var(BMI_*) indep(trueage_*) model(cnorm) min(8) max(52) order(3 3 2 3)

trajplot, xtitle(Age) ytitle(BMI) ci xlabel(76(2)86) ylabel(10(5)45)

tab _traj_Group

traj, var(MAP_*) indep(trueage_*) model(cnorm) min(40) max(170) order(3 3 3)

trajplot, xtitle(Age) ytitle(MAP) ci xlabel(76(2)86) ylabel(85(5)120)

tab _traj_Group

traj, var(PP_*) indep(trueage_*) model(cnorm) min(0) max(170) order(3 3 3)

trajplot, xtitle(Age) ytitle(PP) ci xlabel(76(2)86) ylabel(40(10)90)

tab _traj_Group
